# Tyre rubber exposure causes oxidative stress and intracellular damage in the Baltic clam (*Macoma balthica*)

**DOI:** 10.1007/s11356-025-35893-8

**Published:** 2025-01-22

**Authors:** Pinja Näkki, Aino Ahvo, Raisa Turja, Erika Sainio, Arto Koistinen, Samuel Hartikainen, Sirpa Peräniemi, Milda Stankevičiūtė, Janina Pažusienė, Kari K. Lehtonen, Outi Setälä, Maiju Lehtiniemi

**Affiliations:** 1 Marine and Freshwater Solutions, Finnish Environment Institute, Latokartanonkaari 11, 00790 Helsinki, Finland; 2 Tvärminne Zoological Station, University of Helsinki, J.A. Palménin Tie 260, 10900 Hanko, Finland; 3 SIB Labs, University of Eastern Finland, P.O. Box 1627, 70211 Kuopio, Finland; 4 School of Pharmacy, University of Eastern Finland, P.O. Box 1627, 70211 Kuopio, Finland; 5 Laboratory of Ecotoxicology, Nature Research Centre, Akademijos St. 2, 08412 Vilnius, Lithuania

**Keywords:** Baltic Sea, Baltic clam, Biomarkers, Cell ultrastructure, Microplastics, Tyre particles

## Abstract

Car tyres are considered to release a substantial amount of particles to the environment. Due to the high emission volumes and the chemical risks associated with tyre rubber, there is an urgent need to quantify their ecotoxicological effects. The effects of exposure to particles derived from end-of-life tyres were investigated on the Baltic clam (*Macoma balthica*), which is one of the key invertebrate species living in the soft-bottom sediments of the northern Baltic Sea. Tyre rubber particles (10–188 µm) were added to the aquaria in an environmentally relevant concentration (1.08 g per kg dry sediment), and the clams had either direct or indirect contact to the particles for 5 and 29 days. The effects of exposure were studied by applying a battery of biomarkers and cell ultrastructure examination of clam tissues, and the concentrations of selected polycyclic aromatic hydrocarbons and trace metals originating from tyre rubber were quantified from the exposure water and clam tissues. The exposure did not affect the mortality of the clams but induced multiple sublethal responses, including an elevated glutathione *S*-transferase activity, a reduction in the activity of superoxide dismutase, and increased oxygen radical absorbance capacity during the prolonged exposure. Macromolecular damage was indicated by elevated cytogenetic damage and ultrastructural changes in mitochondria and lysosomes. The results demonstrate the potential of environmentally relevant concentrations of tyre rubber particles to disturb the health of marine biota and underline their importance as a yet understudied environmental contaminant.

**Supplementary Information:**

The online version contains supplementary material available at 10.1007/s11356-025-35893-8.

## Introduction

Tyre particles are proposed to be one of the largest sources of microlitter in the environment (Lassen et al. 2015; Kole et al. 2017; Setälä and Suikkanen 2020). They are generated from tyres undergoing friction on the road and form agglomerations with the road material, therefore called tyre and road wear particles (TRWP) (Hann et al. 2018). Another significant source of tyre rubber particles is also end-of-life tyres (ELT) that have become waste. Globally, only 15% of the ELTs are estimated to be used for energy recovery, and the remaining proportion may end up to landfills and stockpiles, civil engineering applications, or recycling for further use (Valentini and Pegoretti 2022a, b). Recycling is the preferred option for ELT management: for recycling purposes, the tyres are grinded into particles of various sizes and then reintroduced to the environment in many forms, e.g. in artificial turfs, playground surfaces, or rubber-modified asphalt (Valentini and Pegoretti 2022a, b).

The actual emission loads of TRWP and other tyre-derived particles to the marine environment are still virtually unknown (Wagner et al. 2018), and identification and discrimination of TRWP and ELT particles from the environment are challenging partly due to analytical deficiencies (Magni et al. 2024). In many cases, their abundance has been indirectly assessed from the production volumes of tyres and their calculated emissions during use or by detecting specific chemical markers known to be present in tyres (Halle et al. 2020).

Tyre rubber particles from various sources can be transported to nearby waterways by winds and run-off. When in water, these particles are expected to sink due to being denser than water (density between 1.15 and 1.20 g cm^−3^; reviewed by Halle et al. 2020) and accumulate into sediments; for example, average concentrations of TRWP in the Seine river sediments are relatively high, reaching 4.5 g per kg of dry sediment (Unice et al. 2013). In stormwater detention systems located in the Swedish west coast, the estimated concentrations of tyre-derived particles ranged from < 0.15 to 10.8 g per kg of dry sediment at the different study sites (Wik et al. 2008). Tyres are a complex source of pollutants: the rubber material consists of a mixture of natural and synthetic rubber polymers, including styrene-butadiene rubber (SBR), and various additives, e.g. carbon black, different stabilisers, oils, fillers, and pigments (Halsband et al. 2020; Kole et al. 2017). Tyre rubber contains numerous hazardous substances that can leach out of the material, the most abundant being trace metals (e.g. zinc [Zn], iron [Fe], and cobalt [Co]), benzothiazole, polycyclic aromatic hydrocarbons (PAHs), and phenolic compounds (Capolupo et al. 2020; Celeiro et al. 2014; Councell et al. 2004; Halsband et al. 2020). Lately, a highly toxic quinone transformation product of N-(1,3-dimethylbutyl)-N′-phenyl-p-phenylenediamine) (6PPD) was also discovered, being toxic, e.g. for coho salmon *Oncorhynchus kisutch* at very low concentrations, and deriving from tyre rubber where it is utilised as an antiozonant (Tian et al. 2020).

Comparing the ecotoxicological effects of tyre particles is challenging as the composition of different types of tyres as well as the composition of ELT particles, TRWPs, and laboratory-generated rubber fragments differ (Evans and Evans 2006; Magni et al. 2024). Previously, Magni et al. (2024) have studied the effects of ELT granules and ELT particles on the zebrafish *Danio rerio* in aqueous suspensions (concentrations 0.1, 1, and 10 mg L^−1^), focusing on various endpoints for cellular stress, neurotoxicity, and behaviour and physiology. In their study, alterations in the activities of detoxifying enzymes, swimming behaviour, and heart rate, as well as modulations of proteins were observed. Also, Carrasco-Navarro et al. (2021) found induction of cellular stress causing alterations in the gene expression in *Chironomus riparius* as a response to ELT particle exposure in concentrations reaching 10 mg L^−1^. However, the same material caused no effect on the growth, survival, or reproduction of *C. riparius* and another freshwater sediment-dweller *Lumbriculus variegatus* in the follow-up study utilising concentrations corresponding to 1, 3, and 10% of sediment dry weight (Carrasco-Navarro et al. 2022).

Similarly, laboratory-generated tyre tread particles mixed into the sediment at concentration of 10% of sediment dry weight did not affect the survival and growth of the freshwater benthic invertebrates *Asellus aquaticus*, *Gammarus pulex*, and *Tubifex* spp., or the growth of *L. variegatus* in a 28-day exposure (Redondo-Hasselerharm et al. 2018). In a different study, when micronised tyre rubber fragments were administered to water, they did reduce the growth and reproductive output of the freshwater amphipod *Hyalella aztec*a following a 21-day exposure in concentrations of 0.145 g L^−1^ and 0.29 g L^−1^, respectively (Khan et al. 2019). In the study above, different toxicity profiles for tyre rubber particles and their leachates were found, suggesting that the acute toxicity of the particles is distinct from that of the leached chemicals. However, their modes of toxic action remain unclear, calling upon further comparative research. In addition, since most of the above-mentioned studies were carried out using standard laboratory biotest species, it is important to examine the effects also on species with more ecological relevance.

As many of the conducted studies on tyre rubber focus mainly on acute mortality endpoints and reproductive toxicity, there is a need to investigate sublethal effects in the organisms (Halle et al. 2020). Biomarkers are measurable indicators of molecular, cellular, or physiological changes that reflect the exposure to environmental chemicals or their toxic effects and can be detected relatively quickly after exposure (Peakall 1994). They are widely used in ecotoxicological research as early warning signals of adverse health effects before the impacts can be seen at the level of populations, communities, or whole ecosystems (Lam and Gray 2003). Combining various biomarkers that represent different functions and levels of organisation is a useful tool for evaluating the general health of the organism and, in our study, to gain indication on the mechanism of action of the chemical contaminants present in tyre rubber.

Being exposed to contaminants often increases the formation of reactive oxygen species (ROS), which can provoke oxidative damage to proteins, lipids, and DNA if their intracellular neutralisation becomes inadequate (Davies 1995; Livingstone et al. 1990; Valavanidis et al. 2006). The antioxidant defence system (ADS) of cells scavenges ROS, and the changes in the activity of ADS enzymes can be used as an indicator of developing oxidative stress (de Zwart et al. 1999; Valavanidis et al. 2006). Furthermore, if the ADS is not able to counteract the damage caused by ROS, structural changes in various macromolecules and cell organelles may occur, indicating a more severe stress response (Livingstone 2001).

In the current study, the effects of short-term and prolonged ELT rubber exposure were investigated in one of the key invertebrate species living in the soft-bottom sediments of the brackish-water Baltic Sea, the Baltic clam (*Macoma balthica*). This was done by using a battery of biomarkers and examination of the cell ultrastructure anomalies giving indications of the ADS response and possible oxidative stress and intracellular damage. In addition, a targeted analysis of selected trace metals and PAHs were conducted from the exposure water and from clam tissues to detect the presence of potentially harmful substances in tyre rubber. We hypothesised that direct exposure to tyre rubber would cause negative effects in clams, indicated by the selected biomarkers, and that less effects would be seen in the indirect filtrate exposure trying to imitate the exposure to tyre rubber leachates. Regarding the exposure duration, we hypothesised that the prolonged exposure would result in greater oxidative stress in clams compared to the short-term exposure.

## Materials and methods

The exposure experiment was carried out in two phases, short term (5 days) and long term (29 days). They both consisted of three treatments in separate aquaria: control, filtrate treatment, and particle treatment. In the filtrate treatment, the tyre rubber particles were confined in a mesh bag to mimic indirect exposure, while in the particle treatment, the particles were released directly into the water and left to sink freely to the sediment surface. The following abbreviations are used for different phases and treatments: SC, short-term control; SF, short-term filtrate; SP, short-term particle; LC, long-term control; LF, long-term filtrate; LP, long-term particle.

### Preparing the tyre rubber

The tyre rubber used in the experiments originated from ELTs obtained from a recycling company operating in the EU internal market. The material was mechanically fragmented to 200–500 µm particles and further grinded and cryo-milled (Retsch MM400, Retsch GmbH, Haas, Germany) to produce rubber powder (diameter < 200 μm). The rubber particles were examined using scanning electron microscopy (SEM, Zeiss Sigma HD|VP, Carl Zeiss NTS, Cambridge, UK) to determine their size distribution. The resulting material consisted of irregular rubber fragments (size range 10–188 μm), of which 52% were smaller than 34 µm (Table S1, Figure S1). Nylon mesh fabric (1 µm mesh size) was folded into a two-fold bag (27 cm × 17 cm), and 100 g of tyre rubber powder (estimated to represent an approximate concentration of 1.08 g per kg of dry sediment) was sewn inside using a sewing machine. Double seams were used to prevent the loss of powder from the seams, and the length of the stitch was the smallest possible (0.1 mm). Similar mesh bags without any tyre rubber particles were also prepared for the control and particle treatments.

### Experimental set-up

Natural sediment and Baltic clams for the experiment were collected from the surroundings of the Tvärminne Zoological Station (University of Helsinki) in the Gulf of Finland, northern Baltic Sea, onboard R/V *Saduria* in June 2019. The sediment was collected using a van Veen grab sampler, and the clams were caught using a bottom trawl (site Storfjärden, 59°51.249 N; 23°16.239 E, depth 35 m). The sediment in this area is characterised as fine-grained with the dominant fractions consisting of silt and clay (< 63 µm), and fine and very fine sand (63–250 µm) (e.g. Näkki et al. 2019, 2017).

A 1-mm sieve was used for removing resident macrofauna from the sediment. The retained sediment was left overnight to settle in buckets, and on the next day, the overlying water was removed. The sediment was homogenised, and 35 L was added to each aquarium (length 60 cm, width 50 cm, height 50 cm); eventually, the sediment settled as an 11-cm-thick layer. The aquaria were placed in a climate-controlled room (10 °C, 14:10 light–dark cycle), 60 L of seawater (salinity 5–6) was added to fill the aquaria, and constant aeration was established. The aquaria were left to settle for 4 days before starting the experiment.

Callipers were used to measure the length of the clams selected for the experiment (16–18 mm). Only clams moving their siphons or foot were selected to ensure that they were alive and in a good condition. Six hundred clams were acclimatised in an aquarium containing ambient seawater (10 °C, dissolved oxygen 7.12 mg/L, salinity 5–6) with constant aeration for 4 days before starting the experiment and fed with live *Nannochloropsis* (Phytomaxx, NYOS® Aquatics, Germany). After the acclimatisation period, 100 clams were distributed to each aquarium. A glass jar (height 16 cm, diameter 14 cm) containing the mesh bag was placed to the middle of each aquarium; for control and particle treatments, an empty mesh bag was added, and in the filtrate treatments the mesh bag contained tyre rubber particles. For adding tyre rubber particles freely to the SP and LP aquaria, 100 g of rubber powder was pre-mixed with 300 mL seawater. To keep the particles in suspension, 180 µL of the common surfactant Tween20 (Sigma-Aldrich) was also added in all aquaria, also the controls; the resulting concentration (3 µL/L) falls below the no observed effect concentration (NOEC) (7 µL/L) of the substance (Beiras et al. 2018).

Temperature, dissolved oxygen, and pH were measured from the aquaria every other day during the experiment (YSI Environmental ProODO™, PMU 6100 Multiparameter). On the same days, 30 L of seawater was renewed from every aquarium by siphoning the water from the aquarium and filtering it through a 20 µm plankton net. All tyre rubber particles left in the net from the particle treatments were rinsed back into the aquarium to maintain a stable particle concentration throughout the experiment. Thirty L of ambient seawater was then added without disrupting the sediment. After each water renewal, the clams were fed with 2 mL of the algal concentrate (Phytomaxx, NYOS® Aquatics, Germany).

Water samples for trace metal analysis were taken on experiment days 1, 3, 5, 17, and 29 and for the PAH analysis on days 3, 5, and 29. These sampling days were selected to cover the assumed highest concentrations during the experiment (day 3 before the first water renewal) and the end concentration in both experiments (day 5 for the short-term phase and day 29 for the long-term phase). On the third day of the experiment, water samples were taken both before and after the water renewal to assess the change in PAH and trace metal concentrations between the renewals. Additional water samples from the aquaria were taken weekly to monitor the concentration of ammonia (NH_4_-N).

When ending the experiment, all the clams were collected from the aquaria and their mortality was documented. Living clams from each treatment were randomly divided into groups for the different analyses: three clams were dissected for cell ultrastructure examination, 40 clams were dissected for biomarker analysis, 15 clams were used to determine a morphometric condition index and trace metal concentrations in soft tissues, and 27–31 clams were used for PAH analysis in soft tissues.

### Sample processing

#### Elemental analysis from water

Water samples (10 mL) for elemental analysis were taken from each aquarium and from the ambient seawater into 15 mL centrifuge tubes, fixed immediately with 100 µL of 65% nitric acid (Suprapur, Merck), and stored at 4 °C until further analysis. Elemental and trace metal (Zn, Fe, Co, lithium [Li], sodium [Na], magnesium [Mg], aluminium [Al], kalium [K], calcium [Ca], vanadium [V], chromium [Cr], manganese [Mn], copper [Cu], arsenic [As], rubidium [Rb], strontium [Sr], barium [Ba], lead [Pb], selenium [Se], boron [B], and phosphorus [P]) concentrations were determined using inductively coupled plasma mass spectrometry (ICP-MS). Solution analysis was done using a NeXION 350D instrument (PerkinElmer Inc., Waltham, MA, USA) equipped with an ESI PrepFAST autosampler (Elemental Scientific, Omaha, NE, USA) operated in collision mode with kinetic energy discrimination (KED). Two internal standards, yttrium-89 and lutetium-175, were mixed online with the samples to compensate for matrix effects and instrument drift. Analytes were determined against certified multi-element calibration standards (TraceCERT® Periodic table mix 1 for ICP, Sigma-Aldrich) in 2.5% HNO_3_ (TraceMetal™ grade, Fisher Chemical). The samples were diluted within calibration ranges with HNO_3_ solution before ICP-MS measurements. The data processing was conducted with PerkinElmer Syngistix Data Analysis Software™.

Concentrations of sulphur (S), chlorine (Cl), and bromine (Br) were measured by total reflection X-ray fluorescence spectrometry (TXRF) using gallium (TraceCERT® 1000 mg/L Ga standard for AAS in 5% HNO_3_, Sigma-Aldrich) as an internal standard. The S2 Picofox TXRF instrument (Bruker, Germany) was used for measurements and Spectra software for data analysis.

#### Analysis of PAHs from water

Approximately 500 mL of water was also taken from each aquarium to brown glass bottles and stored at 4 °C until analysis in the FINAS-accredited Metropolilab Oy Ab (Helsinki, Finland). A total of 24 individual PAH congeners were analysed: naphthalene (NP), 2-methylnaphthalene (2-MNP), 1-methylnaphthalene (1-MNP), biphenyl (BP), 2,6-dimethylnaphthalene (2,6-DMN), acenaphthylene (ACY), acenaphthene (ACE), 2,3,5-trimethylnaphthalene (2,3,5-TMN), fluorene (FLU), phenanthrene (PHE), anthracene (ANT), 1-methylphenanthrene (1-MPH), fluoranthene (FLA), pyrene (PYR), benzo[a]anthracene (BaA), chrysene (CHR), benzo[b]fluoranthene (BbF), benzo[k]fluoranthene (BkF), benzo[e]pyrene (BeP), benzo[a]pyrene (BaP), perylene (PER), indeno[1,2,3-cd]pyrene (IcdP), dibenzo[a,h]anthracene (DahA), and benzo[g,h,i]perylene (BghiP).

The extraction and analysis of PAHs from the experiment waters were conducted corresponding to ISO/TS 28581 (ISO, 2012). Briefly, a mixture of internal standards (acenaphthene-d10, phenanthrene-d10, chrysene-d12, perylene-d12, naphthalene-d8) and extraction solvent (40 mL of hexane) were added to the weighted sample bottle. The contents of the bottle were mixed for 30 min in a shaker (GFL 3016, Germany). After mixing, the solvent phase was separated from the water in a funnel. Na_2_SO_4_ was added to the extract and shaken for 20 min in a shaker. The dried extract was evaporated first to 1 mL in TurboVap II (Biotage, UK) and then to 0.1 mL with a stream of nitrogen gas. The extract was analysed using gas chromatography–mass spectrometry: 7890A GC (Agilent Technologies USA) – 7000B Triple Quadrupole (Agilent Technologies USA) and analytical column Agilent HP5-MSUI, 30 m × 0.25 mm × 0.25 um).

#### Analyses of trace metals from clam soft tissues

Trace metal analyses were conducted in the FINAS-accredited laboratory of the Finnish Environment Institute (Helsinki, Finland). The freeze-dried clams were grouped into two replicates from each treatment, and the analysis of the following metals was carried out: As, Cd, Co, Cr, Cu, Fe, Ni, Pb, Se, U, and Zn.

A single reaction chamber microwave digestion unit (Ultrawave, Milestone S.r.l., Sorisole, Italy) was employed in the acid digestion of the samples. A 15-vessel sample rack and 15 mL acid leached quartz vessels were used. Approximately 0.3 g of lyophilized sample was weighed accurately in the vessels, and 6 mL of conc. 67–69% HNO_3_ (Super Purity grade, ROMIL Ltd., Cambridge, England, UK) and 0.3 mL of internal standard solution were added. Before starting the programme, a base load of 120 mL of water and 8 mL of 30% H_2_O_2_ (Super Purity grade, ROMIL Ltd., Cambridge, England, UK) was added to the chamber. The sample rack was placed in the chamber after which it was closed and pressurised (40 bar). A temperature-controlled microwave programme with a maximum power of 1500 W was set up consisting of a 20-min ramp to 240 °C followed by a 15-min dwell time. After running the programme and cooling and depressurizing the chamber, the samples were transferred into acid leached polypropylene test tubes and diluted to 30 mL with deionized water. The samples were diluted 10× with deionized water prior to elemental analysis.

Elemental analysis was carried out using ICP-MS (Thermo iCAP Q, Thermo Fisher Scientific, Bremen, Germany). Scandium (Sc), gallium (Ga), rhodium (Rh), and iridium (Ir) were used as internal standards. To remove spectral interferences, a collision cell utilising kinetic energy discrimination (KED) was used with helium as collision gas. Three reference samples were digested and measured along with the samples, NRC DORM-4 Fish Protein (National Research Council of Canada), IAEA-461 *Gafrarium tumidum*, and IAEA-476 Fish Homogenate (International Atomic Energy Agency, Monaco). Uranium was the only element not certified in any of the reference materials. All other results were in good agreement with the certified values, with yields ranging from 87 to 109%. Blank samples were digested with every sample batch, and the results were below the limit of quantification.

#### Analysis of PAHs from clam tissues

For the PAH analyses, all the remaining clams (27–31 from each aquarium) were measured with callipers, removed from their shells, placed in glass vials, and stored at − 20 °C. From each treatment, clams were pooled to form two or three replicates to provide sufficient biomass for the analysis (two replicates from all long-term treatments LC, LF, LP; three replicates from short-term treatments SC, SF, SP). A total of 21 PAH congeners were analysed: NP, 2-MNP, 1-MNP, ACY, ACE, FLU, PHE, ANT, FLA, PYR, BaA, triphenylene (TRI), CHR, BbF, BkF, BeP, BaP, PER, IcdP, DahA, and BghiP. Depending on the congener, the limit of quantification (LOQ) was 1–2 µg per kg ww.

The pooled clam samples were homogenised with an IKA Ultra-Turrax homogenizer T18 (Staufen, Germany). Each sample was weighed (2.6–8.5 g) into a Falcon tube, and 10 µL of internal standard (ISTD) was added to the samples. The samples were shaken vigorously for 15 s and left to stand at room temperature for 15 min. Ten millilitres of UHQ water and 10 mL of ethyl acetate (EtOAc) were added into the tubes and shaken for 1 min. Buffered salts (4 g MgSO_4_ and 2 g NaCl) were added and shaken vigorously for 1 min followed by centrifugation (4000 rpm, 15 min). The EtOAc phases were collected, evaporated to dryness, and reconstituted in 1 mL of isooctane. Samples were purified using Phenomenex Strata-1 SPE cartridges (1 g, 6 mL). The PAH compounds were eluted with 2 × 5 mL *n*-hexane. Extracts were evaporated to 2 mL followed by the addition of 1 mL of isooctane. Finally, the samples were concentrated to a volume of 0.5 mL.

Chemical analysis of PAH compounds was performed using a gas chromatography-tandem mass spectrometer (GC–MS/MS). The GC–MS/MS analysis was conducted with a Thermo Scientific Trace 1310 GC system (Milan, Italy) coupled to a Thermo Scientific TSQ Ultra mass spectrometer (San Jose, USA) in electron impact (EI) (+ 70 eV) mode. The analysis was done in the MRM mode. A six-point calibration (ca. 2–2500 pg/μL) was applied. Then, 1 μL of each extract was injected using split injection (split ratio 1:10). The GC oven temperature was programmed as follows: 70 °C (1 min), 60 °C/min, 210 °C, 5 °C/min, 250 °C, 15 °C/min, 280 °C, and 325 °C (10 min). The transfer line temperature was 300 °C. The separation was achieved by implementing a Select PAH column (Agilent Technologies). A duplicate analysis was performed for every pooled sample. Blank and control samples were analysed to ensure the performance of the instrument.

#### Dissection of gills, foot, and digestive gland tissues of the clams for biomarker analyses

A total of eight biochemical biomarkers were determined from the digestive gland: reduced glutathione (GSH) and oxidised glutathione (GSSG) ratio (GSH/GSSG); the activities of glutathione reductase (GR), catalase (CAT), glutathione peroxidase (GPx), superoxide dismutase (SOD), and glutathione *S*-transferase (GST) enzymes; and the degree of lipid peroxidation (LPO) and oxygen radical absorbance capacity (ORAC). In addition, the foot was used for the measurement of acetylcholinesterase (AChE) activity, a neurotoxicity indicator, and the gills for the total cytogenetic damage (combined geno- and cytotoxicity) analyses. In addition, a morphometric condition index (CI) was determined using the shell length vs. soft tissue dry weight relationship.

The soft tissue of clams (*n* = 40 from each aquarium) was removed from the shells, and the gill, foot muscle, and digestive gland tissues were dissected. For geno- and cytotoxicity analyses, gill samples were collected from 20 specimens from each treatment. Small pieces of gills were dissected, and the preparation of slides and investigations was performed utilising the methods described in Baršienė et al. (2006, 2004). The foot tissues of two individuals were pooled to form one sample to obtain enough material for the analyses, and the digestive glands were individually stored. Both tissue sample types were immediately frozen in liquid nitrogen and stored at − 80 °C. The maximum length of the shell was measured with a calliper.

#### Sample homogenization

Digestive gland samples of 20 clams from each treatment were homogenised individually in 100 mM potassium phosphate buffer (pH 7.4) and centrifuged at 10,000 *g* for 20 min at 4 °C for CAT, GST, GR, GPx, and SOD analyses. For AChE, foot samples (pooled from two individuals) were individually homogenised in 100 mM Na-PO_4_, (pH 7.0), containing 0.1% Triton-X100, and centrifuged at 10,000 *g* for 20 min at 4 °C. All the supernatants were stored at − 80 °C.

Samples for LPO, GSH/GSSG, and ORAC from the remaining 20 individuals were homogenised in 100 mM potassium phosphate buffer with 150 mM sodium chloride (pH 7.4). For the LPO analysis, samples of the homogenate were taken before centrifugation and 4% BHT (in methanol) was added to inhibit peroxidation. The samples were centrifuged at 10,000 g for 20 min at 4 °C. Samples for ORAC were obtained from the supernatant after the centrifugation and stored at − 80 °C. Then, 20 µL of the remaining supernatant was incubated on ice for 10 min with 40 µL of 5% SSA (sulfosalicylic acid). Then, the samples were centrifuged at 10,000 g for 10 min at 4 °C. Aliquots of the supernatant were stored at − 80 °C with 3 mM M2VP (1-methyl-2-vinlypyridinium triflate) for GSSG and without the chemical for GSH.

#### Biomarker measurements

Enzyme activity rates of CAT, GST, GR, GPx, SOD, and AChE; protein concentrations of the homogenate; and the amount of LPO were measured in 96-well half-area plates with a microplate reader (Infinite 200, TECAN) and Magellan software (TECAN). The reaction rate was evaluated according to the best linearity range of the curves. The enzyme activities were adjusted to the protein concentrations of the samples and determined on microplates following the Bradford (1976) method and a bovine serum albumin standard. ORAC, GSH and GSSG, and LPO were measured in 384-well plates with a TECAN Spark spectrophotometer. The samples were measured in triplicate or quadruplicate.

##### ADS enzymes and total antioxidant capacity

The activity of CAT in the digestive gland was determined following Claiborne (1985) and Vuori et al. (2015). In brief, the activity is measured as the change in absorbance in a mixture containing 4.3 μM H_2_O_2_ at a final concentration in phosphate buffer and expressed as µmol min^−1^ mg protein^−1^. GPx in the digestive gland was determined according to Vuori et al. (2015) utilising the commercial kit Sigma CGP1-1KT, modified for digestive gland samples. In brief, the GPx activity is measured as the change in absorption at 340 nm in a mixture containing 0.5 mM NADPH, 4.2 mM GSH, and 1 unit/mL GR at a final concentration in a NaN_3_ (sodium azide) supplemented reaction buffer. Activity of GR in the digestive gland samples was measured using the methods by Vuori et al. (2015). Briefly, the activity of GR is measured as the change in absorbance at 340 nm in a mixture containing 1 mM GSSG, 0.75 mM DTNB, and 0.1 mM NADPH at a final concentration in EDTA-phosphate buffer (100 mM K-PO_4_ + 2 mM EDTA, pH 7.5). GPx and GR activities are expressed as nmol min^−1^ mg protein^−1^. For measuring SOD, a commercial kit (Merck 19160) was used. SOD activity is expressed as U (units) min^−1^ mg protein^−1^. GSH and GSSG were measured using a commercial kit (Arbor Assays Detect X Glutathione Fluorescent Detection Kit, Catalog No K006-F5) and expressed as µM mg protein^−1^ and as their ratio (GSH/GSSG). ORAC measurements were done utilising the commercial kit OxiSelect Oxygen Radical Antioxidant Capacity (ORAC) Activity Assay (Cell Biolabs STA-345) and expressed as µM of Trolox (the standard) equivalents [TE] mg protein^−1^.

##### Oxidative damage

LPO levels were measured as thiobarbituric acid reactive substances (TBARS) at 535 nm (Ohkawa et al. 1979). The reaction mixture consisted of 60 mM Tris–HCl with DTPA, 0.24 M trichloroacetic acid, and 16 mM 2-thiobarbituric acid incubated for 60 min at 95 °C. LPO is expressed as nmol TBARS g ww^−1^.

##### Detoxification

The activity of GST in the digestive gland was measured following the methods by Habig et al. (1974), modified for microplate: the activity of GST is measured as the colour change in 340 nm in a mixture containing 2 mM GSH and 1 mM CDNB (1-chloro-2,4 dinitrobenzene) at final concentration in Dulbecco’s buffer. GST activity is expressed as nmol min^−1^ mg protein^−1^.

##### AChE activity

The method described by Bocquené and Galgani (1998) was used for measuring AChE activity. In brief, the AChE activity is measured as the colour change at 412 nm in a mixture containing 0.5 mM DTNB (5,5′-dithiobis 2-nitrobenzoic acid) and 2.6 mM ACTC (acetylthiocholine iodide) at a final concentration in phosphate buffer. AChE activity is expressed as nmol min^−1^ mg protein^−1^.

##### Geno- and cytotoxicity

Stained slides were analysed with bright-field Olympus BX51 microscope (Tokyo, Japan) equipped with an immersion objective (1000 ×). A total of 2000 cells with intact cellular and nuclear membranes were evaluated per each clam by using blind scoring. The final results are expressed as the mean value (‰) of the sums of the analysed individual lesions scored in 1000 cells per individual sampled from every treatment. Induction of micronuclei (MN), nuclear buds on filament (NBf), nuclear buds (NB), blebbed nuclei (BL), and bi-nucleated cells with nucleoplasmic bridges (BNb) were evaluated as genotoxicity endpoints, and induction of fragmented apoptotic (FA), bi-nucleated (BN), and 8-shaped nuclei cells were assessed as cytotoxicity endpoints. Due to the low frequencies of 8-shaped nuclei and BN cells, these parameters were summed up and expressed as total cytotoxicity. The total genotoxicity level is expressed as the sum of all analysed genotoxicity endpoints (MN + NBf + NB + BL + BNb); together with the total cytotoxicity, they express the level of total cytogenetic damage. Nuclear abnormalities were identified using pre-described criteria (Baršienė et al. 2014; Fenech et al. 2003; Heddle et al. 1991).

##### Condition index

A morphometric condition index (CI) was calculated for 15 individuals from each treatment. The soft tissue was removed from the shell, rinsed carefully with clean seawater, placed individually in Eppendorf tubes, and stored at − 20 °C. A calliper was used to measure the maximum length of the shell, and the soft tissues were freeze-dried for 24 h at − 60 °C in 10^−1^ atm (SuperModulyo freeze dryer, Thermo) and weighed (Mettler AT250). CI was determined as tissue dry weight (mg) /shell length (mm) ^3^ × 100 (Bonsdorff and Wenne 1989; Sokolowski et al. 1999).

#### Cell ultrastructure analysis of the gill, foot, and digestive gland tissues

At the end of the experiment, three clams from each treatment were dissected for the cell ultrastructure examination. The soft tissue was removed from the shell, and the gills, foot, and digestive gland were dissected and placed in the fixative droplets (3% glutaraldehyde in 0.1 M sodium cacodylate buffer). Inside the droplet, 1 × 1 mm pieces of tissue were cut with a scalpel and the pieces were stored in Eppendorf tubes containing the same fixative at 4 °C.

The specimens were prepared by post-fixation in 1% osmium tetroxide with cacodylate buffer solution at 4 °C for 1 h. Once rinsing with cacodylate buffer, the specimens were dehydrated in graded ethanol (70, 90, 94, and 100%) and infiltrated and embedded in epoxy resin. An ultramicrotome was used to cut 1 µm sections, which were stained with 1% toluidine blue for light microscopy examination. The sections were examined with a Zeiss AxioImager M2 (Carl Zeiss Microscopy GmbH, Jena, Germany), and representative sites were chosen for electron microscopy (EM) analysis. For EM, ultrathin Sects. (70 nm) were again cut with an ultramicrotome and stained using 1% uranyl acetate and lead citrate. Observations of the epithelial cells of each tissue type were conducted at a voltage of 200 kV with a JEOL JEM-2100F transmission electron microscope (Jeol Co, Tokyo, Japan) equipped with a digital camera Quemesa (Olympus-SIS, Münster, Germany). A blinded observer examined the tissue sections and reported the apparent findings in the epithelial cells (e.g. structural changes and damage in the organelles). The findings were merged with the experimental setup, and the effects of exposure were concluded. Qualitative differences were reported similarly to, e.g. Korkalainen et al. 2017.

### Statistical analyses

Regarding chemical analyses, concentrations below the LOQs were assumed to be at the quantification limit; thus, they represent maximum estimates of PAH concentrations in water and in clams in the experiment.

Statistical analyses were done with R (v. 4.0.3; R Core Team 2020), using the car package (v. 3.0–8, Fox and Weisberg 2019) and the PMCMRplus package (v. 1.6.1, Pohlert 2020). A one-way analysis of variance (ANOVA) with Tukey’s post hoc test was applied to test the differences in biomarker responses between the treatments. Levene’s test was used to verify the homogeneity of variances, and Shapiro–Wilk test was used to confirm normality. When the assumption of equal variances was not fulfilled, Welch ANOVA with a Games-Howell post hoc test was applied, and log_10_ transformation was used to resolve deviations from normality. If the transformation did not correct the normality, a non-parametric Kruskal–Wallis test was used, followed by the pairwise Wilcoxon rank sum test with BH adjustment.

A significance level of 0.05 was used, and the results are given as the mean ± standard deviation (SD). The figures were drawn with R (v. 4.0.3; (R Core Team 2020) using packages ggplot2 (v. 3.3.2, (Wickham 2016), gridextra (v.2.3, (Auguie and Antonov 2017)), and cartocolor (v.1.0.0, (Nowosand 2018) and edited with Inkscape (v. 1.0).

## Results

### Experimental conditions

Temperature, dissolved oxygen, and pH in the aquaria remained constant during the experiment (9.42 ± 0.63 °C; 10.62 ± 0.50 mg/L; 7.86 ± 0.05, respectively). The NH_4_-N concentration was 1149.28 ± 81.40 µg/L before the first water renewal (day 3) and after that 667.28 ± 39.92 µg/L for the rest of the first experiment week. During the following weeks, it stabilised to 239.52 ± 50.87 µg/L. The mortality of clams in the aquaria was on average 3.2% (± 1.3), 3.5% ± 0.7 during the long-term exposure (LF and LP), 2% ± 1.4 in the short-term exposure (SF and SP), and 4% ± 1.4 in the controls (SC and LC).

### Elemental concentrations in water

Concentrations of some chemical elements (B) and metals (e.g. Li, Na, Mg, K, and Ca) were similar in all aquaria and resembled those measured in the seawater control. Their concentrations also remained relatively stable or showed a slightly decreasing trend during the experiment (Table S2). The concentrations of some other elements (P) and trace metals (e.g. Al, Fe, and Ba) showed an increasing trend but were finally quite similar in all long-term treatments at the end of the experiment. Some of the elements had low concentrations in the seawater control but exhibited the highest ones in all treatments at the start of the experiment and then gradually declined similarly in all the aquaria (e.g. V, Ni, As, and Rb); these elements likely had a sedimentary origin.

However, concentrations of some trace metals (e.g. Co, Cu, and Zn) fluctuated during the experiment, also showing variability between the treatments. The most noticeable were the concentrations of Zn in the particle treatments SP and LP (692–706 μg/L on day 3 before water renewal) compared to the controls SC and LC (2.6–6.5 μg/L), filtrate treatments SF and F (7.3–17.5 µg/L), and to the seawater control (16.3 ± 9.6 µg/L). A relatively high concentration of Zn was also quantified in LF (100 µg/L) compared to LC (18.2 µg/L) and LP (26.8 µg/L) at the end of the experiment. In addition to Zn also, Co showed elevated concentrations in the particle treatment aquaria SP and LP on day 3 (2.7–3.0 µg/L) compared to all the other aquaria (0.4–0.6 µg/L) and the seawater control (0.08 ± 0.02 µg/L), being still elevated in the SP aquarium at the end of the experiment. A similar trend was also seen regarding Cu, which showed concentrations of 5.0–6.8 µg/L in the SP and LP treatments on day 3, whereas lower ones were measured in the other treatments and the seawater control (1.7–2.2 µg/L and 2.5 ± 0.9 µg/L, respectively).

### PAH concentrations in water

**Fig. 1:**
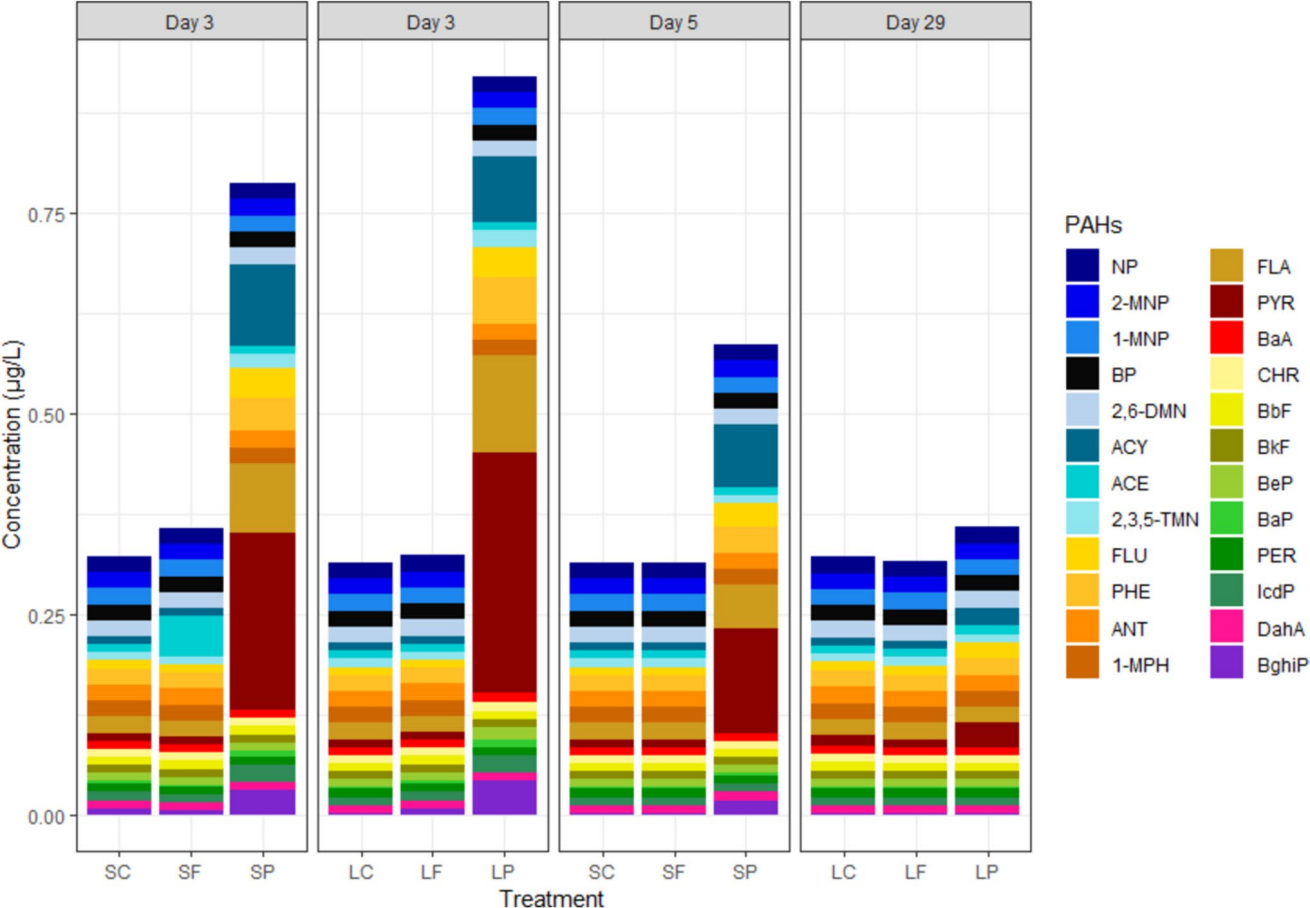
PAH concentrations in the experiment water. Maximum concentrations of individual PAH congeners in water in the different treatments based on their LOQs on different sampling days (SC, short-term control; SF, short-term filtrate; SP, short-term particle; LC, long-term control; LF, long-term filtrate; LP, long-term particle). PAHs presented in the figure: naphthalene (NP), 2-methylnaphthalene (2-MNP), 1-methylnaphthalene (1-MNP), biphenyl (BP), 2,6-dimethylnpahtalene (2,6-DMN), acenaphthylene (ACY), acenaphthene (ACE), 2,3,5-trimethylnapthalene (2,3,5-TMN), fluorene (FLU), phenanthrene (PHE), anthracene (ANT), 1-methylphenanthrene (1-MPH), fluoranthene (FLA), pyrene (PYR), benzo[a]anthracene (BaA), chrysene (CHR), benzo[b]fluoranthene (BbF), benzo[k]fluoranthene (BkF), benzo[e]pyrene (BeP), benzo[a]pyrene (BaP), perylene (PER), indeno[1,2,3-cd]pyrene (IcdP), dibenzo[a,h]anthracene (DahA), and benzo[g,h,i]perylene (BghiP)

The highest concentrations of PAHs in water were recorded in the particle treatments SP and LP (Fig. 1, Table S3). In these treatments, they exceeded their LOQs in the case of the following congeners: 2,6-DMN, ACY, 2,3,5-TMN, FLU, PHE, FLA, PYR, CHR, BeP, BaP, IcdP, and BghiP. The most abundant PAHs in SP and LP were PYR (0.27–0.30 µg/L), FLA (0.098–0.120 µg/L), and ACY (0.065–0.100 µg/L) on day 3, after which, their concentrations decreased. Some PAHs (e.g. FLU, PYR, BaP, IcdP, and BghiP) were occasionally over their LOQs in control aquaria and probably originated from the seawater and the sediment used in the experiment. However, most PAHs were below their LOQs and hence the sum of PAHs was very similar between SC, SF, LC, and LF.

### Trace metals in clam soft tissues

**Fig. 2:**
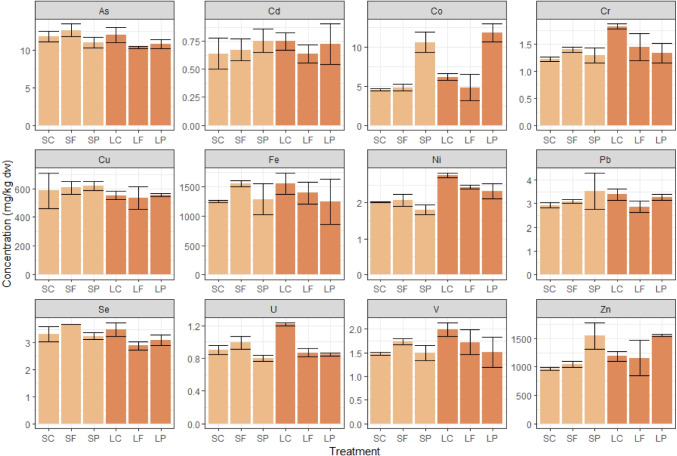
Trace metal concentrations in clams. Concentrations (± standard deviation) of trace metals in clams in the different treatments at the end of the experiment, expressed as mg/kg dry weight (SC, short-term control; SF, short-term filtrate; SP, short-term particle; LC, long-term control; LF, long-term filtrate; LP, long-term particle). Note the different scalings of the *y*-axes

For most trace metals, the concentrations analysed in the clam soft tissues did not differ between the treatments (Fig. 2). However, the mean Zn and Co concentrations were clearly elevated in the SP and LP treatments (Zn 1549–1555 mg/kg dw; Co 10.6–12 mg/kg dw) compared to SF and LF (Zn 1047–1154 mg/kg dw; Co 4.8 mg/kg dw) and SC and LC (Zn 961–1188 mg/kg dw; Co 4.5–6.2 mg/kg dw) (Fig. 2, Table S4).

### PAHs in clam soft tissues

**Fig. 3:**
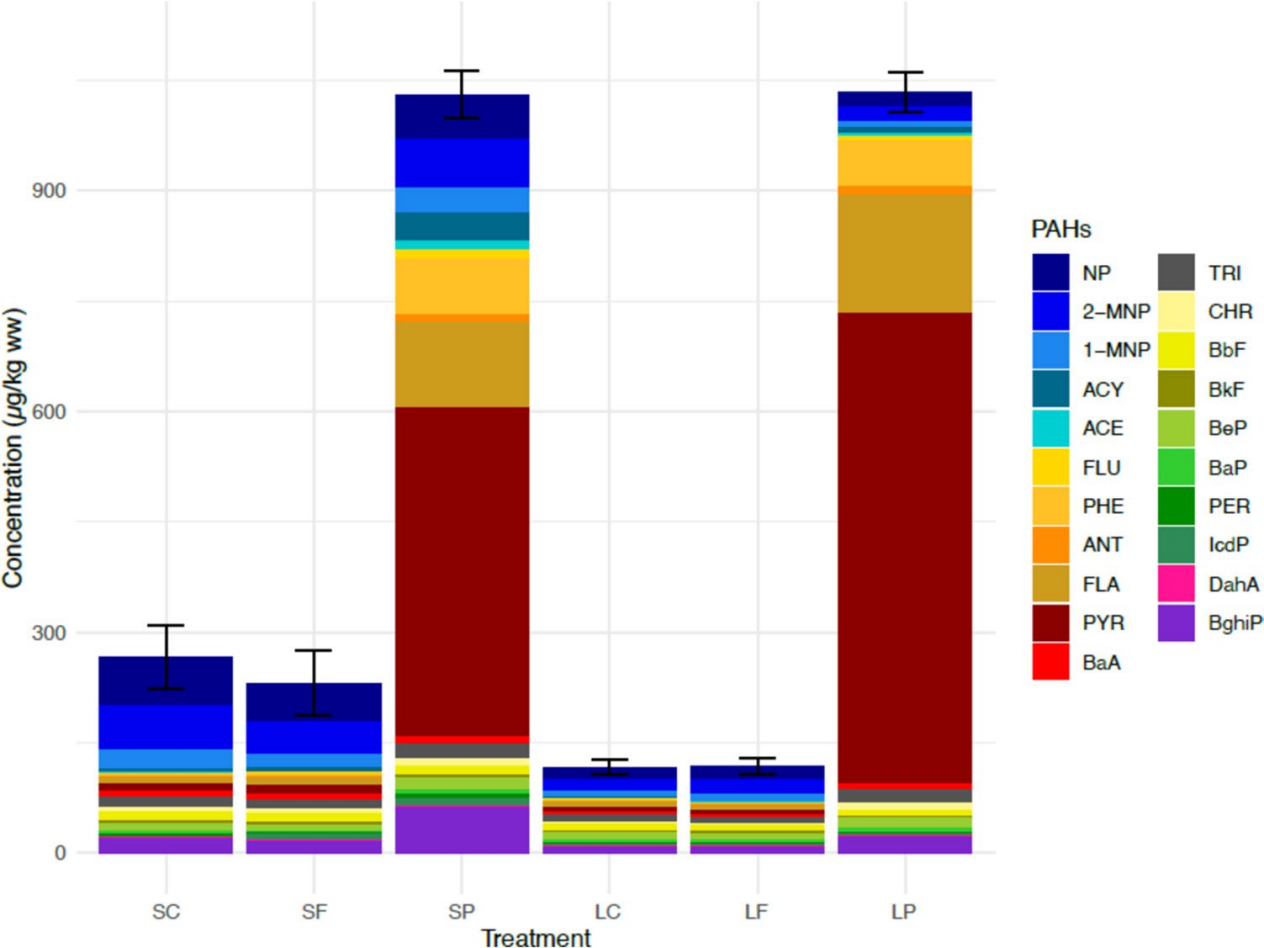
PAH concentrations in clams. Maximum concentrations of individual PAH congeners in the soft tissues of clams at the end of the experiment based on their LOQs in the different treatments and the standard deviation of the sum of PAHs (SC, short-term control; SF, short-term filtrate; SP, short-term particle; LC, long-term control; LF, long-term filtrate; LP, long-term particle). PAHs presented in the figure: naphthalene (NP), 2-methylnaphthalene (2-MNP), 1-methylnaphthalene (1-MNP), biphenyl (BP), 2,6-dimethylnpahtalene (2,6-DMN), acenaphthylene (ACY), acenaphthene (ACE), 2,3,5-trimethylnapthalene (2,3,5-TMN), fluorene (FLU), phenanthrene (PHE), anthracene (ANT), 1-methylphenanthrene (1-MPH), fluoranthene (FLA), pyrene (PYR), benzo[a]anthracene (BaA), chrysene (CHR), benzo[b]fluoranthene (BbF), benzo[k]fluoranthene (BkF), benzo[e]pyrene (BeP), benzo[a]pyrene (BaP), perylene (PER), indeno[1,2,3-cd]pyrene (IcdP), dibenzo[a,h]anthracene (DahA), and benzo[g,h,i]perylene (BghiP)

On average, the clams in the particle treatments SP and LP contained highest PAH sums (1029 µg/kg ww and 1033 µg/kg ww, respectively) (Fig. 3). The most abundant PAHs in both particle treatments were PYR, FLA, and FLU. While the sum of PAHs and the dominant congeners were similar in the SP and LP treatments, the concentrations of some individual PAHs varied: for example, NP, 2-MNP, ACE, ACY, PER, and BghiP were higher in the SP treatment. In the LP treatment, FLA and PYR were relatively more abundant than in the SP treatment. The sum of all PAH congeners in clams in the SC and SL treatments was very similar to each other as well as in clams in the LC and LF treatments. When comparing the short-term and long-term exposures, the sum of PAHs was higher in the SC and SF treatments (SC 265 µg/kg ww; SF 230 µg/kg ww) compared to the LC and LF treatments (LC 116 µg/kg ww; LF 117 µg/kg ww).

### Biomarker responses

**Fig. 4:**
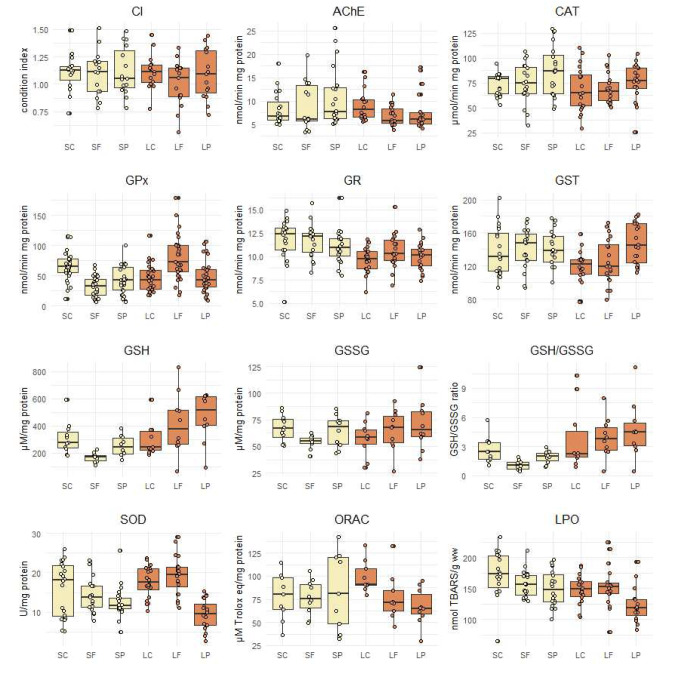
Biomarkers. Biomarkers measured in clams in the different treatments at the end of the experiment (SC, short-term control; SF, short-term filtrate; SP, short-term particle; LC, long-term control; LF, long-term filtrate; LP, long-term particle)

**Fig. 5:**
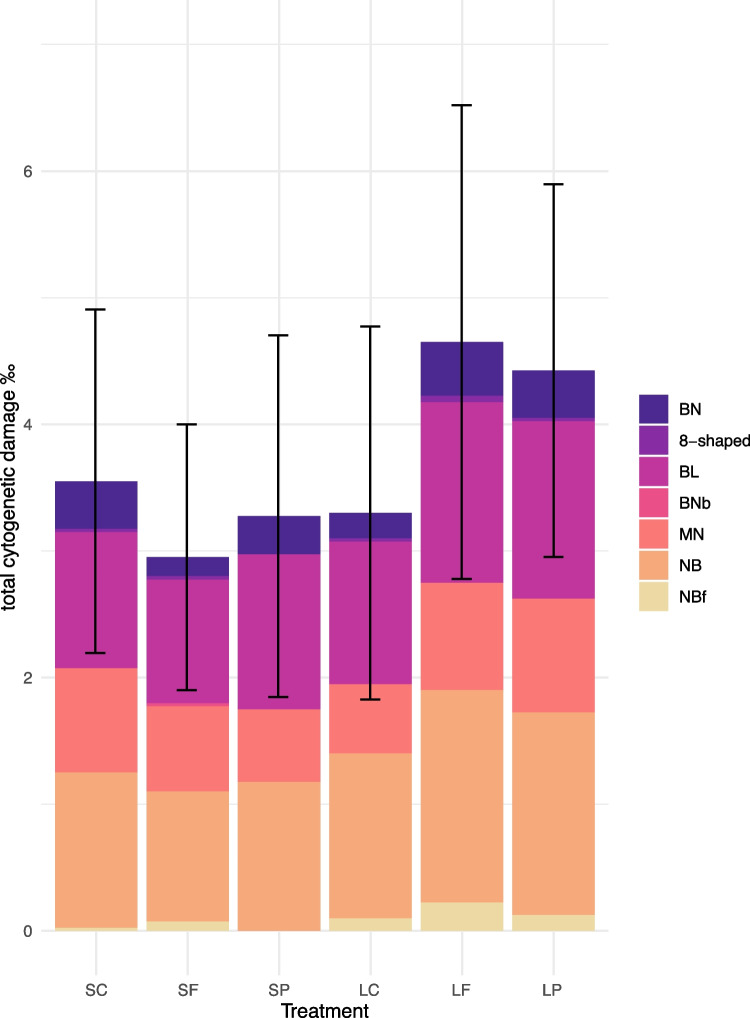
Total geno- and cytogenetic damage. Total geno- and cytogenetic damage (± standard deviation) in clams in the different treatments at the end of the experiment (SC, short-term control; SF, short-term filtrate; SP, short-term particle; LC, long-term control; LF, long-term filtrate; LP, long-term particle). Bi-nucleated (BN) and 8-shaped nuclei cells describe cytotoxic effects and the rest represent genotoxic effects (BL, blebbed nuclei; BNb, bi-nucleated cells with nucleoplasmic bridges; MN, micronuclei; NB, nuclear buds; NBf, nuclear buds on filament)

Most of the measured biomarkers showed significant differences between the treatments (Table 1, Fig. 4) except for AChE, CAT, and GSSG. The combined genotoxicity and cytotoxicity (i.e. total cytogenetic damage) was elevated and close to being significantly different (*p* = 0.056) in the LF and LP groups compared to LC (Fig. 5, Table S6). No significant differences in mean CI of clams between the different treatments could be detected; however, variability between the individuals was notably higher in both exposure groups compared to the control group both in the short-term and long-term treatments (Fig. 4).

**Table 1:** Statistical tests. Statistical tests performed on each biomarker in different treatments (ANOVA with Tukey’s HSD multiple comparisons, Welch ANOVA with Games-Howell multiple comparisons, or Kruskal–Wallis with pairwise Wilcoxon rank sum test and BH adjustment) are shown below each biomarker. The significance is indicated in bold (*p ≤ 0.05, **p ≤ 0.01, ***p ≤ 0.001). The significant differences found in pairwise comparisons are shown on the right side of the table. Treatment codes: SC, short-term control; SF, short-term filtrate; SP, short-term particle; LC, long-term control; LF, long-term filtrate; LP, long-term particle

		SC	SF	SP	LC	LF
GPx	SF	0.000***				
H(5) = 44.701, *p* = 0.000***	SP	0.003**	0.135			
	LC	0.003**	0.046*	0.874		
	LF	0.165	0.000***	0.000***	0.000***	
	LP	0.008**	0.053	0.874	0.874	0.001***
GR	LC	0.002**	0.004**	0.077		
F(5, 113) = 5.25, *p* = 0.000***	LP	0.015**	0.031*	0.311	0.986	0.833
ORAC	LP	0.897	0.853	0.850	0.012*	0.931
F(5, 22.912) = 3.0404, *p* = 0.03*						
SOD	LC	0.444	0.096	0.004**		
F(5, 52.499) = 14.274, *p* = 0.000***	LF	0.478	0.012*	0.000***	0.853	
	LP	0.011*	0.009**	0.145	0.000***	0.000***
LPO	LP	0.000***	0.023*	0.105	0.174	0.061
F(5, 103) = 4.889, *p* = 0.000***						
GSH	LF	0.961	0.008**	0.501	0.901	
F(5, 55) = 4.886, *p* = 0.000***	LP	0.497	0.000***	0.098	0.380	0.942
GSH/GSSG	SF	0.039*				
F(5, 55) = 5.405, *p* = 0.000***	LC	0.996	0.012*	0.685		
	LF	0.881	0.002**	0.333	0.992	
	LP	0.613	0.000***	0.137	0.899	0.997
GST	LP	0.832	0.938	0.951	0.004**	0.056
F(5, 113) = 3.912, *p* = 0.003**						
Total cytogenetic damage	LF	0.123	0.013*	0.056	0.056	
H(5) = 18.692, *p* = 0.002**	LP	0.091	0.013*	0.056	0.056	0.910

### Examination of the cell ultrastructure

**Fig. 6:**
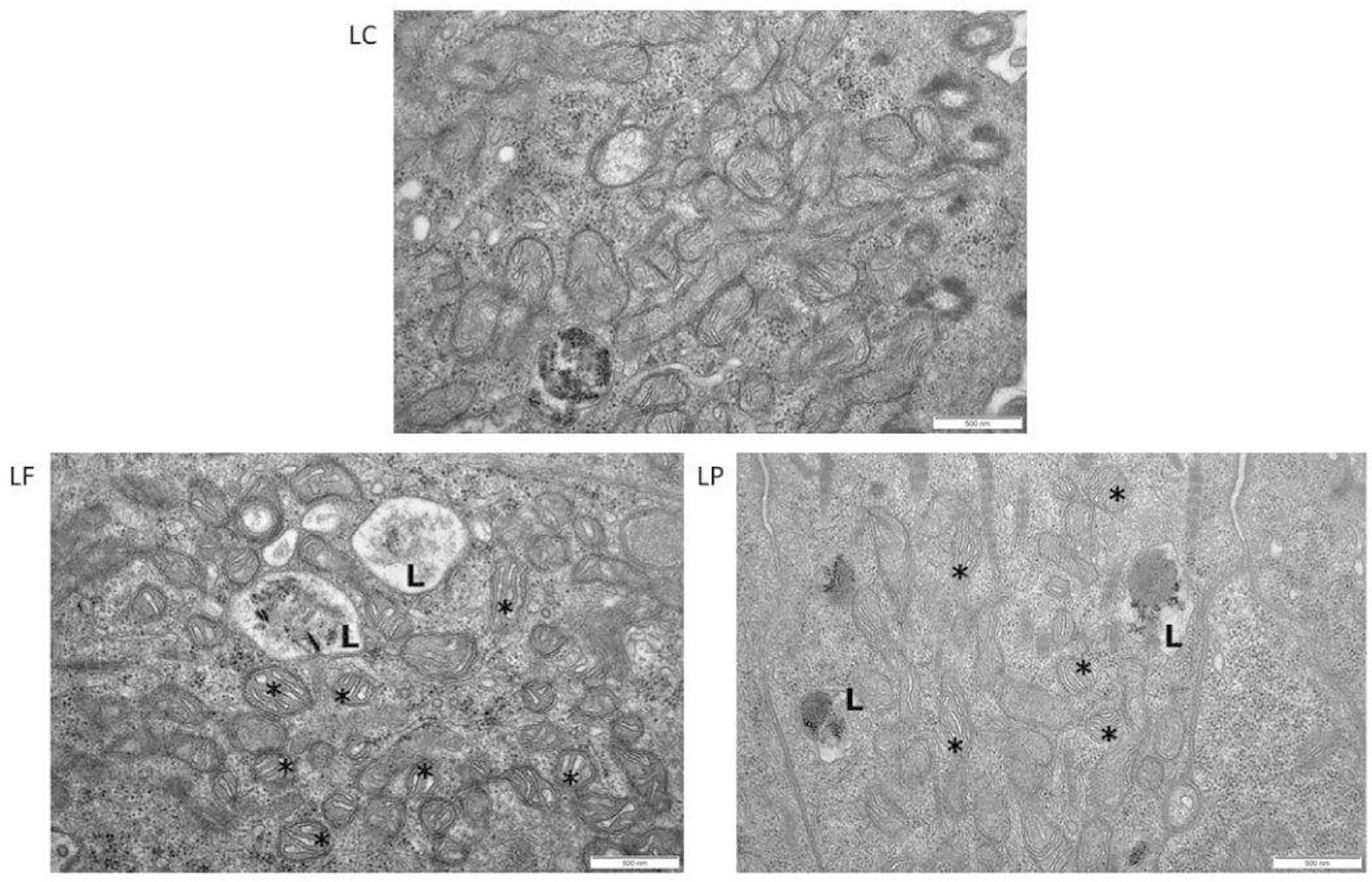
Ultrastructural changes. EM photos (15 000 × magnification) from the gills (LC, long-term control; LF, long-term filtrate; LP, long-term particle). In the LF and LP sections, swollen mitochondria (marked with asterisk) and lysosomes (marked with L) are visible

Most deviations from the normal cell ultrastructure occurred in the treatments SP, LF, and LP, where changes were observed in all the examined clam tissues (Table S7). Notable changes were seen especially in the epithelial cells of the gills of SP, LF, and LP clams: loosened cell junctions, increased lysosomal number and size were observed in the SP group, whereas clams in the LF and LP treatments exhibited swollen mitochondria (Fig. 6). In addition, the lysosome number and size were also increased in the LF group and the lysosomes contained dark material in both LF and LP groups (Fig. 6). This may indicate the activity of the tissues to remove or degrade hazardous components or damaged organelles. In contrast, the gill tissue of clams in the SF and LC treatments exhibited overall normal cell structure (tight junctions, lysosomes, and cilia); only in the SF group the mitochondria were slightly swollen.

Considering the digestive gland samples, clams in the SC and SF groups appeared to be normal. However, uneven cilia on the epithelium were observed in SP, LC, LF, and LP groups and swollen mitochondria in the LIC and LP treatments. Inside the cells, the number of lysosomes was increased in LC and LP clams, suggesting cellular damage or degradation. In the foot tissue, the epithelium had an even structure in all the examined clams and there were less changes compared to the gills. Inside the cells, the structure of the mitochondria appeared normal in the LC, SF, and LF groups. The foot tissue samples in SC, SP, and LP treatments were similar to each other and showed a decreased number of mitochondria, an increased number of lysosomes, and also dark material inside them.

## Discussion

### Trace metals and PAHs in tyre rubber and clam soft tissues

Elevated levels of some trace metals and many PAHs were detected in the water samples in both short-term and long-term particle treatments SP and LP compared to the control and filtrate treatments. The origin of some of the metals (e.g. Zn, Co, and Cu) and PAHs (e.g. PYR, FLA, and ACY) could be traced back to the tyre rubber, based on the substantial differences in their concentrations in the particle treatments compared to the seawater control and the variability in their concentrations between the different treatments and timepoints. This interpretation is further supported by previous studies that have also identified these substances from tyre rubber. For example, studies conducted specifically with ELT particles, Zn and Cu were observed in the particles and their leachates (Lehtiniemi et al. 2021; Magni et al. 2022, 2024). Despite the fact that the chemical ingredients of tyre rubber can vary depending on the brand of tyres as well as the type of tyre particles studied, high concentrations of Zn and Co are commonly detected, as well as many PAH congeners, especially FLA, PYR, and BghiP also in studies focusing on other tyre-derived particles and their leachates (e.g. Capolupo et al. 2020; Celeiro et al. 2014; Halsband et al. 2020). As in the present experiment, the water samples were analysed unfiltered, and the concentrations represent both the contaminants leached from tyre rubber to the water as well as those present in the material itself. The trace metal and PAH concentrations in the water samples decreased over time, probably due to the combination of periodic water renewals, the settlement of the smallest tyre rubber particles in the water phase, and their subsequent burial by bioturbation.

In the particle treatments SP and LP, the contaminant profiles in clam tissues reflected the profiles detected in the water samples; elevated concentrations of, e.g. Zn and Co, and PYR and FLA, were found. This similarity is likely a result of tyre rubber ingestion by the clams. During the experiment, the incurrent siphons of the clams were seen to draw tyre rubber particles inside the siphons, and tyre rubber was also observed inside the clams’ soft body mass during the dissection and digestive gland homogenization. Since all the soft tissues of the clams were used for the metal and PAH analyses, they inevitably contained tyre rubber; hence, it is in this kind of an experimental set-up impossible to separate the contaminant load of the rubber particles from the potential accumulation of the leached contaminants into the clam tissues.

Interestingly, the congener profiles were slightly different in SP and LP clams: more low molecular weight (LMW; 2–3 aromatic rings) PAHs (NP, 2-MNP, 1-MNP, ACY, ACE, FLU, and PHE), but also BgiP, were found from the SP clams, whereas in LP clams, FLA and PYR were more abundant. As the concentrations of the lightest PAHs (NP, 2-MNP, 1-MNP) were elevated also in SC and SF compared to the LC and LF, they may have been present in the sediment at the start of the experiment and diluted during the many water renewals. Also, some of the LMW PAHs (e.g. ACY and ACE) may have also quickly desorbed from the tyre rubber during the first days of the experiment, leading to their higher concentrations in clams in the SP treatment. The potential change in the PAH profile of tyre rubber with time may indicate that its effects on biota could depend on the age of the rubber material. Also, earlier studies investigating the effects of tyre rubber exposure have observed reduced toxicity following a sequential leaching of the test material (Hartwell et al. 1998; Wik et al. 2009), also suggesting that leaching of contaminants from the rubber material decelerates with time.

Regarding the filtrate exposure, since neither the water samples nor the clam tissues showed differences in trace metal and PAH concentrations between the controls and filtrate treatments, the leaching of these contaminants from tyre rubber and their subsequent bioconcentration in the clams could not be verified. It is possible that the stationary experimental setup (mesh bag in a glass jar) prevented efficient leaching, as the particles formed a cluster inside the mesh bag instead of being well dispersed in the water. Previously, it has been observed that the leaching of Zn is more pronounced from smaller ELT particles, probably due to the increased surface/volume ratio of particles (Magni et al. 2022), so the clustering of the particles in the current experiment may have reduced the surface/volume ratio needed for efficient leaching. This is further supported by the observation that the concentrations of some contaminants (e.g. Zn in day 29; Table S2) in water samples from the LF treatment elevated towards the end of the experiment when the bag was gently swayed in the water, allowing more space between the enclosed rubber particles. However, it is not known whether this truly enhanced the leaching or possibly could have released tiny tyre rubber fragments into the water. Hence, some caution must be taken when interpreting the results obtained from the filtrate treatments.

### Biomarkers

While the observed differences between the particle treatments and the other treatments were clear in terms of tyre rubber-related contaminant profiles and concentrations, the measured biomarkers showed varying responses. In the present study, AChE did not show any significant differences between the treatments, which is line with the study conducted with *D. rerio* and ELT particles by Magni et al. (2024). In previous studies with other bivalve species, exposure to polystyrene microplastics has resulted in contradicting responses and either inhibited the activity of AChE (Ribeiro et al. 2017), caused no effect at all (Magni et al. 2018), or the response has varied depending on the studied tissue (Avio et al. 2015).

A highly variable response pattern was observed in the ADS response. This is a widely observed phenomenon since practically all the single parameters are mechanistically interlinked, also regarding the time and duration of exposure. Many antioxidant enzymes exhibit a bell-shape response where the highest activities of the enzymes occur at moderate stress and exposure levels, and severe stress leads to decreased activity due to the inability to produce the ADS proteins as a bioenergetic trade-off in favour of other vital mechanisms to maintain cellular homeostasis.

In the SF and SP treatments, both SOD and GPx showed reduced activity, indicating extensive stress due to the exposure resulting in inability to respond by the increased production of these enzymes. The total oxyradical scavenging capacity measured as ORAC shows also extensive individual variability in the SP group. GSH is a non-enzymatic antioxidant agent that protects the cellular membranes against lipid peroxidation by actively scavenging ROS. In the short-term experiment, the GSH/GSSG ratio was lower in SP treatment and significantly reduced in the SF treatment compared to the control group. This signifies an increased need and utilisation of GSH in scavenging ROS and for Phase II detoxification (conjugation) and the subsequent depletion of the GSH pool.

The long-term experiment indicates adaptive metabolic rearrangements. Neither the levels of GSSG or GSH nor the activity of GR, the enzyme that replenishes GSH from its oxidised form GSSG, showed any significant differences between the treatments, also suggesting an adaptive response. A significantly higher GST activity was observed in the LP treatment compared to the control treatment, indicating increased detoxification. Subsequently, the significantly lowered SOD activity in the LP treatment compared to the control treatment (and also to the LF treatment) suggests a bell-shape response in this enzyme; the clams have already been exposed for nearly a month, and the physiological capacity of individuals in the LP group has been diminished during this time, causing the reduction of SOD activity and, thus, the measured low activity. ORAC shows decreased levels, this time in both exposure treatments but significantly in the LP group, confirming a decline in the capacity to deal with the increased production of ROS under the long-term exposure. Conclusively, this condition is likely to lead to macromolecular damage and consecutively to various health effects and reduced fitness in case the exposure continues. Interestingly, and also confirming the mechanistic paradigm that moderate exposure increases enzymatic ADS biomarker activities, GPx shows highly elevated activity in the LF group compared both to the control treatment and to the LP treatment, which are at the same level but for completely different reasons (see discussion above).

All in all, the results from the long-term experiment are well in line with the chemical measurements in the LP and LF treatments, and the biomarkers applied in the present study could detect exposure in both cases. Finally, the level of LPO in the LP treatment is near to being significantly lower compared to that in the LC and LF treatments and significantly lower than that in the SC and SF treatments in the short-term exposure. This observation suggests that the ADS response has been able to protect the cell membranes during long-term exposure. However, there is no certainty how long this protection mechanism could continue to be effective if the exposure were more prolonged.

The biomarker response observed in the particle treatments is connected to the temporal dynamics of the exposure consisting of the administration of the tyre rubber materials to the aquaria, exposure time, and rate of potential bioaccumulation of the measured contaminants in clam tissues. Of the measured time points, the concentrations of total PAHs in the exposure media were highest at day 3 and had already markedly reduced by day 5. Subsequently, extensive amounts of PAHs were detected in clams during this period in the SP treatment, most notably due to increased levels of HMW congeners PYR and FLA (4 rings) and also FLU (3 rings). The rapid uptake of tyre rubber fragments and potentially occurred subsequent bioaccumulation of especially these PAHs may have led to the clear ADS response consisting mainly of the depletion of GSH and reduction in the baseline production of protective enzymes, i.e. the bell-shape response at high exposure concentrations. When comparing SP (day 5) and LP (day 29) practically, no change could be observed in total PAH concentrations in tissues, and only small differences in the congener profiles were visible (e.g. the levels of LMW 1-MNP and 2-MNP showed a slight reduction). Metabolization of PAHs in bivalves is widely known to be very slow (therefore, they are used in biomonitoring of these substances); thus, it is suggested that during this period, a steady state like dynamics between the continuing uptake of PAHs via tyre rubber ingestion and detoxification took place in the exposed clams. Conclusively, the marked alterations in the ADS response pattern in clams in the LP treatment after the 29-day exposure compared to the 5-day exposure represent adaptive response mechanisms for cellular protection. However, it should be noted that although the exposure time in the long-term experiment was markedly longer, a major part of the contamination was distributed as a pulse-type of exposure directly during the first (short-term exposure) part of the study. Thus, the responses observed later on by day 29 must be considered as a combination of recovery from the extensive contaminant pulse and exposure to gradually decreasing contaminant levels.

The increased level of total geno- and cytogenetic damage detected in LF and LP treatments is suspected to be caused by the increased formation of ROS in response to tyre rubber already during the earlier part of the experiment. However, by day 5, no effects could yet be detected between the treatments, but the initiation of the damage could have already started during this early phase when the exposure concentrations were the highest and the ADS system was still not adapted to the situation. At day 29, we may be seeing the changes caused by the earlier events, or what is left of them, since the stability of the damaged structures is uncertain. To the best of our knowledge, effects of either tyre rubber or more specifically, ELT particles, on genotoxic and cytotoxic endpoints in aquatic invertebrates have not been investigated earlier. To evaluate cytogenetic damage caused by tyre rubber, several in vitro studies have been performed with laboratory-generated tyre particles or TRWP (e.g. Gualtieri et al. 2008, 2005; Karlsson et al. 2011). Studies have reported genotoxicity and cytotoxicity and oxidative stress in human A549 cells (Beretta et al. 2007; Gualtieri et al. 2008, 2005), genotoxicity and inflammatory response in macrophage and pulmonary epithelial cell lines (Karlsson et al. 2011, 2006; Lindbom et al. 2007, 2006) associated with exposure to tyre particles or their extracts. Therefore, toxic effects of tyre rubber particles on aquatic organisms are poorly understood, and the mechanism underlying their potential genotoxic and cytotoxic activity has not been elucidated. Based on the results presented here, the elevated cytogenetic damage levels in *M. balthica* may be related to the oxidative potential of ELT rubber as a complex mixture of chemicals or to certain chemical compounds present, e.g. PAHs and trace metals, or chemicals not quantified in this study. Many PAHs and their metabolites are of particular concern in genotoxicity due to their toxic, mutagenic, and carcinogenic features (Baršienė et al. 2012). Generally, chemical compounds that cause damage to DNA can act directly on the DNA, generate metabolites that provoke DNA damage, increase the production of ROS, or hinder the synthesis and repair of DNA (Lee and Steinert 2003). Thus, the genotoxic risk associated with tyre rubber as a carrier of mixtures of contaminants remains unclear and needs further investigations.

Clams exposed to the tyre rubber filtrate did not bioaccumulate PAHs or trace metals and in many cases showed lesser biological responses compared to those in the particle treatments. However, the observed significant changes in GPx activity and the glutathione system as well as the detected cell ultrastructural differences and cytogenetic damage may indicate the presence of other leached chemicals. Earlier studies have shown that tyre rubber also releases many organic compounds such as benzothiazole and BHT used as antioxidants and vulcanization additives, phthalate plasticizers and phenols (Capolupo et al. 2020; Celeiro et al. 2014; Li et al. 2010; Llompart et al. 2013), and multiple transformation products of 6PPD, including the 6PPD-quinone (Seiwert et al. 2022; Tian et al. 2020), none of which were analysed in the present experiment. It is also notable that Khan et al. (2019) found different toxicity profiles for tyre rubber particles and leachates, suggesting that the chemicals responsible for the toxic effects may be different in tyre rubber particle and leachate exposures. In the study above, the leachates induced higher acute toxicity for the amphipod *Hyalella azteca* under low exposure conditions, whereas in high concentrations, the particles elicited a higher toxic effect. In addition, Magni et al. (2024) observed that the toxicity of ELT suspensions was primarily associated to the chemicals released from the material. In respect to these findings, further research is needed to shed light on the toxic effects caused by exposure to tyre particles and their leachates.

The present experiment showed that small ELT fragments contain and release extensive amounts of PAHs when introduced to brackish water, and these apparently act as major toxic components causing at least part of the responses observed in the clams. Studies on PAH exposure on biochemical biomarkers in *M. balthica* are not available. In another sediment-dwelling clam, *Astarte borealis* from the Arctic, Szczybelski et al. (2019) recorded no effects on AChE, GST, or acyl-CoA oxidase exposed to sediments contaminated by PAHs (range 0.2–1.7 mg/kg dw), suggesting that the contaminant levels used were too low to induce detectable biomarker responses, although metabolites of PAHs were observed in tissues, indicating uptake of the parent compounds. Likewise, no effect on total oxygen scavenging capacity (TOSC) was found by Camus et al. (2003) in another sediment-associated bivalve, *Mya truncata*, exposed to crude oil contaminated sediment in an Arctic field experiment, suggesting that cold temperatures might lead to specific adaptive responses. However, in many other studies, ADS responses similar to those detected here have been recorded in bivalves (mostly mussels of the genus *Mytilus*) in exposures to single PAH compounds (Magara et al. 2018), single PAHs in combination with other substances (e.g. Chen et al. 2018; Turja et al. 2014), and mixtures of PAHs or different types of oil (e.g. Turja et al. 2020), in most cases related to the exposure concentration and time. Regarding field studies on *M. balthica*, elevated CAT and GST activities were observed along a pollution gradient in the Archipelago Sea (Baltic Sea) (Lehtonen et al. 2006), although not related specifically to PAH pollution.

Although the tyre rubber exposure elicited multiple changes in the ADS, the general condition of the clams, as indicated by the CI, did not differ between the treatments. As the CI reflects the nutritional status of the clams (Byrne and O’Halloran 2000), the results suggest that there was enough food available in the aquaria and that the exposure to tyre rubber did not markedly affect the feeding rate or energy metabolism of the clams during the experimental period. Furthermore, in earlier experimental study on the effects of tyre-derived particles on the clam *Scrobicularia plana*, no effects in the CI were seen during the 21-day exposure despite the concentrations were higher than in our experiment (max. 5% of tyre particles in kg of dry sediment; Garrard et al. 2022). Also, in many other previous studies on various bivalve species spanning from 2 weeks to 2 months, no differences have been recorded in CI following microplastic exposure (Bour et al. 2018; Bråte et al. 2018a; O’Donovan et al. 2018; Ribeiro et al. 2017; Sussarellu et al. 2016; Urban-Malinga et al. 2021). However, a reduction in CI was found in juvenile oysters (*Crassostrea gigas*) after an 80-day exposure to the highest tested concentration (10^6^ particles/L) of PS beads (Maes et al. 2020), and a reduction in body condition of the mussel *Semimytilus algosus* after more than 60 days of exposure to high concentrations (150 mg/L) of PVC particles (Barkhau et al. 2022). It is possible that longer experimental periods and juvenile life stages are needed to elicit changes in the CI as it is known that juvenile individuals are more vulnerable to pollution and environmental stress (e.g. recurring hypoxia) than adults (Pineda et al. 2012; Villnäs et al. 2019).

Conclusively, the results show a clear impact of ELT rubber exposure on the ADS and genotoxic and cytotoxic endpoints of *M. balthica* with significant effects still detectable after 1 month from the start of the experiment. In general, only a few studies performed in the aquatic environment have quantified oxidative stress caused by the exposure to ELT particle suspensions or filtrates (Lehtiniemi et al. 2021; Magni et al. 2024), and a handful of studies exists focusing on oxidative stress responses of other tyre-derived particles or their leachates (LaPlaca et al. 2022; Shin et al. 2022; Garrard et al. 2022; Rigano et al. 2024). While the ADS system responses vary considerably from species to species and regarding the type of exposure in question, in general, the above-mentioned studies indicate that both ELT rubber and other tyre-derived particles are able to cause various sublethal effects on a diversity of aquatic taxa ranging from zooplankton to fish. In studies conducted specifically with ELT, Magni et al. (2024) did not observe any significant modulation of the ADS enzymes in *D. rerio* larvae even at the highest tested concentration (10 mg L^–1^), although the detoxifying performance of the enzymes EROD and GST was impacted. However, Lehtiniemi et al. (2021) used ELT particles of the same origin as in the current study to generate a filtrate (30 g L^–1^), which was found to induce significant responses in ADS enzymes of the brackish water copepod *Limnocalanus macrurus* in 72 h exposure. Also in their study, they also gained indication that the ROS formation exceeded the detoxification capacity of the ADS, which seems to be in line with our study detecting oxidative damage in various cell organelles.

### Cell ultrastructure anomalies

Structural changes in various macromolecules and cell organelles may occur if the ADS is not able to neutralise the harmful effects of ROS (Livingstone 2001). Consistently with the described PAH and trace metal concentrations as well as the ADS responses, most cell ultrastructural changes were observed in treatments SP, LIC, and LP. As further evidenced by the elevated levels of total geno- and cytogenetic damage in clams in the LF and LP groups, clustering of all these effects in the same treatments indicates that the prolonged oxidative stress caused by the tyre rubber also resulted in oxidative damage.

In the present study, epithelial cells in the gills of clams in the LF, LP, and SF treatments contained swollen mitochondria. It is known that ROS-induced ruptures in the outer membrane of mitochondria can impair their functions and disturb their volume homeostasis, leading to swelling (Chatterjee et al. 2009; Ott et al. 2007; Slimen et al. 2014). In addition to gills, swollen mitochondria occurred in the digestive gland cells of clams in the LF and LP treatments as well as a decreased number of mitochondria in the foot tissue in the SC and SP treatments. Similar responses have been shown in exposure studies using many environmental contaminants: for example, tributyl chloride caused swelling and alteration of cristae in the blood cells of the rainbow trout *Oncorhynchus mykiss* (Tiano et al. 2003). Also, zinc oxide nanoparticles have been shown to cause swollen mitochondria and loss of cristae in the gills of the Pacific oyster *Crassostrea gigas* after 24 h of exposure and similar but delayed changes in the digestive gland (Trevisan et al. 2014). Similar to our study, it could not be concluded whether the effects were caused by the particles taken up by the cells or dissolved Zn in seawater during the experiment. In any case, the changes observed in the mitochondria may have critical impacts: as the cell metabolism is dependent on the ATP produced in these organelles, disruptions in their function may have detrimental effects on cell viability and, if severely impaired, eventually trigger cell apoptosis (Ott et al. 2007; Slimen et al. 2014).

Another cell organelle showing recurring damage was the lysosome. Lysosomal responses to various pollutants are most often expressed as increased lysosomal size, reduced membrane stability, and changes in lysosomal contents (Marigómez and Baybay-Villacorta 2003). Occasionally, also the number of lysosomes may increase (Marigómez et al. 1996). In the present study, the membrane stability was not investigated, but variability in the size, number, or contents of the lysosomes was observed especially in the gills of clams in the SP, LF, and LP treatments. Previously, the enlargement of lysosomes in the cells of the digestive gland has shown to be a common response for mussels of the genus *Mytilus* following environmental stress or exposure to trace metal or petroleum hydrocarbon pollution (Cajaraville et al. 1995; Marigómez et al. 2005; Regoli 1992). From the PAH congeners measured, both PHE and FLA (400 μg/L) induce swelling of the lysosomes in the digestive gland cells (Moore et al. 2007); however, since the concentrations of PAHs in the present study were considerably lower, that may explain why swelling was not recorded in the lysosomes in any of the digestive gland samples even though PHE and FLA were among the most abundant PAH congeners detected in the study.

It is hypothesised that while the ADS enzymes form the first tier of defence against oxidative damage, lysosomal autophagy (i.e. degradation of cellular components in lysosomes) provides a second line of defence by removing, e.g. oxidatively damaged macromolecules and organelles (Moore et al. 2006a, b, 2008). Autophagy is upregulated during stress (Moore et al. 2006a, b, 2007), and the dark-coloured material seen in the lysosomes in the samples of this study may represent an endpoint of the peroxidation of autophagocytosed cellular components (Dailianis 2010; Moore et al. 2008). Furthermore, when, e.g. metal-damaged organelles are being degraded, the metals accumulate in lysosomes (Fowler 1987), where they can disturb the normal function and damage the lysosomal membrane (Moore et al. 2006a), leading to lysosome enlargement or swelling, which is typical for a general stress response (Marigómez and Baybay-Villacorta 2003; Moore et al. 2008). In conclusion, it seems that the prolonged oxidative stress led to the damage in DNA and mitochondria, further triggering the activation of the lysosomal-autophagic system trying to protect the cells from increased production of ROS.

### Ecological implications

Tyre rubber constitutes a globally significant source of microlitter (Kole et al. 2017; Wagner et al. 2018). Also, in many countries within the Baltic Sea drainage area, it has been estimated that particles derived from tyre rubber form a significant proportion of all microlitter emissions, although it is uncertain how much of it enters marine ecosystems (Sundt et al. 2014; Essel et al. 2015; Lassen et al. 2015; Magnusson et al. 2016; Setälä and Suikkanen 2020). The abundance and distribution of tyre rubber particles in Baltic Sea sediments are still unknown, but indirect evidence of their presence has been obtained from a field survey in the eastern coast of Sweden, where particles were found inside *M. balthica* (Bråte et al. 2020). In that study, the most dominant type of microplastics was black rubbery fragments, which was hypothesised to have originated from tyres, based on a combination of visual inspection, FTIR spectra, and pyrolysis. In other Nordic countries, also *Mytilus* spp. have been shown to ingest similar tyre rubber fragments (Bråte, et al. 2018a, b; Bråte et al. 2020). While further data on tyre rubber exposure of bivalves and other taxa in the natural environment are scarce, it does not necessarily indicate a lack of exposure but may rather reflect the methodological difficulties in material characterization due to the carbon black component of the rubber the use of spectroscopic methods is hampered (Halle et al. 2020). Being a non-selective feeder, it is likely that *M. balthica* ingests tyre rubber particles if they are available on the sediment surface in a sufficiently small size fraction (< 300 µm) (Gilbert 1977; Self and Jumars 1988), as they do with other microplastics (Näkki et al. 2017). Regarding the present experiment, the sediment was not characterised for the tyre rubber or other contaminants upon collection from the field and hence the initial concentrations present in the sediment remain unknown. However, based on the location of the sediment and *M. balthica* collection site in a nature reserve area far from densely populated cities and results of previous contaminant surveys, the site is not expected to be particularly polluted (e.g. Leiniö and Lehtonen 2005). The Tvärminne area has also frequently been used as a non-contaminated site for collecting organisms for experimental laboratory and field studies (e.g. Turja et al. 2023).

The results of the present study describe various stress responses caused by the exposure to ELT rubber fragments; tyre rubber with a relatively similar chemical composition is likely to be released to the environment, e.g. from the artificial turfs using rubber granulates. However, rubber particles generated from tyres on the road are likely mixed with road material, forming TRWP (Hann et al. 2018). Hence, one potentially important component co-occurring with tyre rubber in the environment was potentially missing from our experiment. It is unknown whether the agglomeration of road material with tyre rubber will cause different effects than the tyre fragments alone, but at least it increases the density of the particles and thus their probability to reach the seafloor quickly after they are emitted to the sea (Hann et al. 2018). The pulse-like exposure in our experiment may represent a situation where stormwater runoff or melting of snow introduces a sudden surge of tyre rubber and other contaminants to the receiving coastal waters. Especially benthic, sedentary fauna living in coastal waters near highly urbanised areas are at risk of being exposed to high concentrations of tyre rubber. Since Hartwell et al. (1998) found that the toxicity of tyre rubber leachates increased with decreasing salinity, quantifying tyre rubber abundance in the brackish Baltic Sea should be coupled with experimental studies in locally occurring salinities to be able to assess the potential risk to the resident fauna.

As the results obtained in the current study clearly indicate that *M. balthica* exposed to ELT rubber fragments for long-term had a reduced capacity to counteract the effects of increased ROS production and, subsequently, exhibited signs of oxidative damage, it is highly possible that this condition would ultimately result in reduced fitness. The fitness reduction arises from a bioenergetic trade-off, where in order to survive, the individual is allocating limited energy resources to the ADS and repairing the damage caused by ROS rather than for growth and reproduction, as shown in various invertebrates (e.g. Lister et al. 2016; Petes et al. 2008). However, studies that have aimed at establishing links between tyre rubber exposure and fitness are extremely scarce. In a recent study by Rigano et al. (2024), exposure to TRWP was shown to decrease fertility and increase mortality of *C. riparius*. Stress can also lead to more subtle changes in behaviour; feeding and burrowing rates of *S. plana* were impaired as a response to the exposure to laboratory-generated tyre particles (Garrard et al. 2022). Burrowing was impaired even in the smallest test concentration (0.2% of tyre particles per kg of dry sediment), which indicates that the current levels of tyre rubber particles in the environment are potentially already causing adverse behavioural effects (Unice et al. 2013). However, in the burrowing trial study conducted at the end of the current experiment, no clear indication of reduced burrowing by the exposed clams could be observed (Figure S2). It is possible that longer duration of the trial and more frequent monitoring interval would have been needed for detecting changes in burrowing. However, as previous studies utilising ELT materials have been shown to induce changes in the organism level, such as impaired swimming activity by *L. macrurus* (Lehtiniemi et al. 2021) and increased swimming activity and heart rate by *D. rerio* (Magni et al. 2024), further attention to the behavioural endpoints is needed to better understand the effects of ELT exposure.

Because the current study focused mainly on biochemical and cellular biomarkers, higher-level effects on the fitness of individuals and populations can only be speculated. However, Villnäs et al. (2019) observed changes in the activities of AChE and GR in *M. balthica* exposed to recurring periodic events of hypoxia and a concurrent behavioural change measured as the reburial rate, providing evidence on the linkage of biochemical level biomarkers and higher-level effects. In the Baltic Sea, *M. balthica* is a key species that often predominates the soft-bottom macrozoobenthos (Nikula et al. 2008), has a central role in bioturbation (Michaud et al. 2006), and also serves as an important prey for many fish and bird species (Aarnio and Bonsdorff 1993; Zwarts and Blomert 1992). Especially in the species-poor northern Baltic Sea, it has a substantial contribution to the functioning of the benthic ecosystems, where its functions cannot be entirely replaced by other benthic community members (Villnäs et al. 2012). Thus, possible alterations in the behaviour or changes in population dynamics of *M. balthica* caused by pollution may potentially have significant effects on the functioning of the ecosystem as a whole.

## Supplementary Information

Below is the link to the electronic supplementary material.

Supplementary file1 (DOCX 3.01 MB )

## Data Availability

The research data can be made available by request.
