# Supplementary material for: Tyre rubber exposure causes oxidative stress and intracellular damage in the Baltic clam (Macoma balthica)
Source: Environ Sci Pollut Res Int. 2025 Jan 22;32(7):3951–74. doi: 10.1007/s11356-025-35893-8 (PMC11836145; doi:10.1007/s11356-025-35893-8)
Supplement: Supplementary file 1 — Supplementary file1 (DOCX 3.01 MB ) [file 11356_2025_35893_MOESM1_ESM.docx]

**SUPPLEMENTARY MATERIAL**

**Table S1.** The size of the tyre rubber particles used in the experiment.

| Diameter (mm) | Number of particles | Content (%) |
| --- | --- | --- |
| 0.01 | 208 | 12.7 |
| 0.022 | 464 | 28.4 |
| 0.034 | 180 | 11.0 |
| 0.046 | 149 | 9.1 |
| 0.058 | 131 | 8.0 |
| 0.069 | 132 | 8.1 |
| 0.081 | 85 | 5.2 |
| 0.093 | 72 | 4.4 |
| 0.105 | 57 | 3.5 |
| 0.117 | 59 | 3.6 |
| 0.129 | 31 | 1.9 |
| 0.141 | 16 | 1.0 |
| 0.153 | 11 | 0.7 |
| 0.164 | 13 | 0.8 |
| 0.176 | 17 | 1.0 |
| 0.188 | 9 | 0.6 |
| sum | 1634 | 100 |


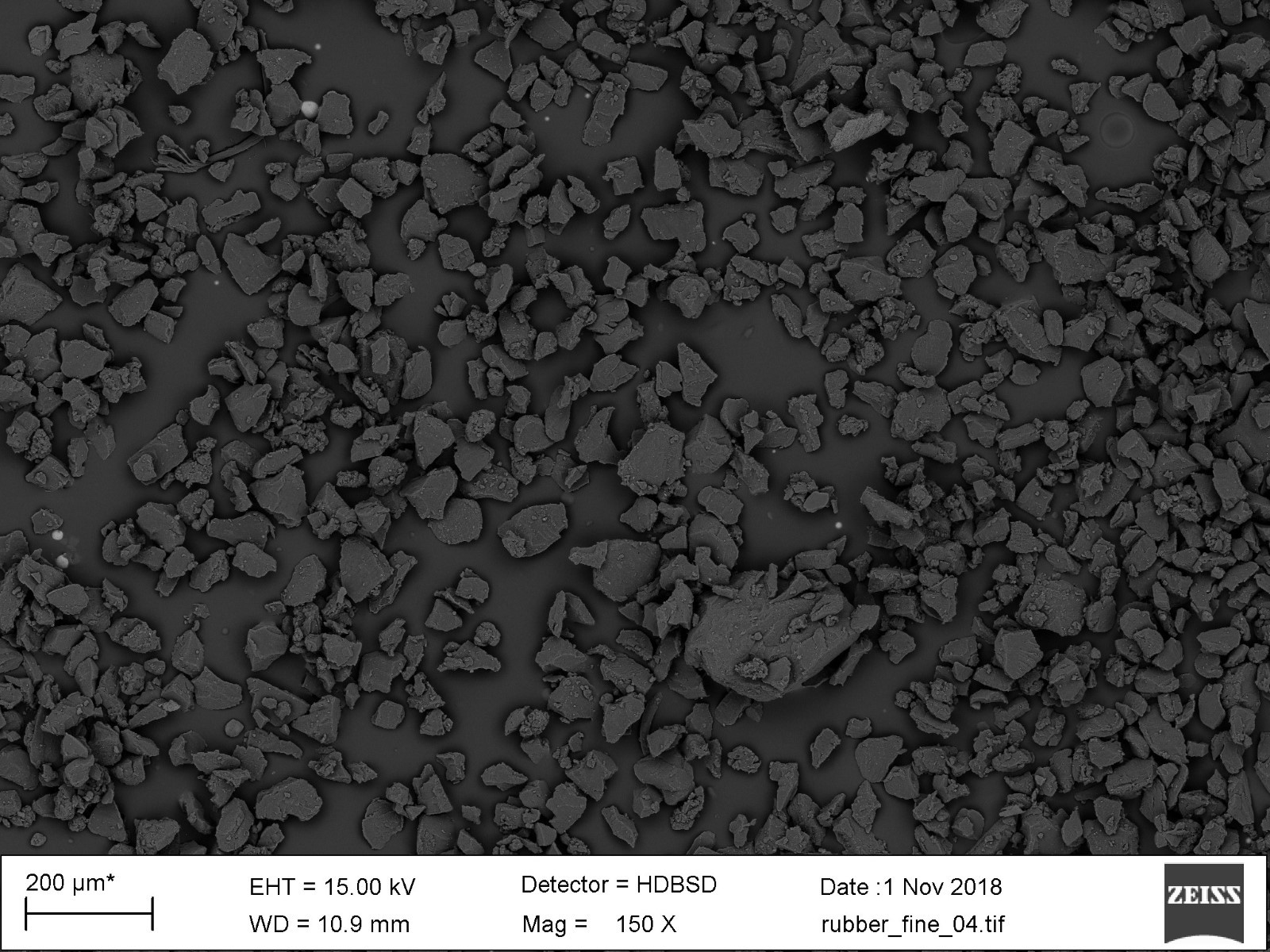


**Figure S1.** Scanning electron microscope photo of the tyre rubber used in the experiment.

**Table S2.** Metal concentrations in the treatment waters and seawater control at different time points (SC = short-term control, SF = short-term filtrate, SP = short-term particle, LC = long-term control, LF = long-term filtrate, LP = long-term particle). At the day 3, sample A has been taken prior to water renewal and sample B after that.

|  |  | ICP-MS |  |  |  |  |  |  |  |  |  |  |  |  |
| --- | --- | --- | --- | --- | --- | --- | --- | --- | --- | --- | --- | --- | --- | --- |
| Treatment | Timepoint | Li 7  (ug/L) | B 11 (ug/L) | Na 23  (g/L) | Mg 24  (mg/L) | Al 27 (mg/L) | P 31 (mg/L) | K 39 (mg/L) | Ca 44 (mg/L) | V 51 (ug/L) | Cr 52 (ug/L) | Mn 55 (mg/L) | Fe 56 (mg/L) | Co 59 (ug/L) |
| SC | start | 58.95 | 1.14 | 2.16 | 258.49 | 2.04 | 0.27 | 98.44 | 54.86 | 8.10 | 3.59 | 0.94 | 0.89 | 0.79 |
| SF | start | 55.97 | 1.10 | 2.06 | 246.26 | 1.49 | 0.33 | 99.53 | 58.81 | 7.47 | 3.06 | 0.89 | 0.69 | 0.67 |
| SP | start | 57.08 | 1.13 | 2.00 | 243.70 | 1.44 | 0.36 | 98.93 | 53.51 | 7.09 | 3.36 | 1.03 | 0.78 | 0.85 |
| LC | start | 50.67 | 0.99 | 2.04 | 241.96 | 1.15 | 0.30 | 94.16 | 55.23 | 5.94 | 3.90 | 0.92 | 0.63 | 0.60 |
| LF | start | 63.09 | 1.12 | 2.00 | 243.52 | 1.47 | 0.44 | 91.19 | 55.47 | 7.20 | 3.06 | 1.07 | 0.76 | 0.77 |
| LP | start | 55.72 | 1.08 | 2.05 | 250.65 | 1.84 | 0.30 | 101.41 | 57.10 | 7.63 | 3.65 | 0.93 | 0.80 | 0.80 |
| SC | Day 3 A | 58.45 | 1.16 | 2.04 | 245.51 | 1.10 | 0.33 | 95.09 | 56.12 | 5.68 | 2.28 | 0.83 | 0.50 | 0.46 |
| SC | Day 3 B | 52.01 | 1.03 | 2.02 | 244.02 | 0.32 | 0.17 | 88.38 | 52.87 | 2.87 | 1.92 | 0.36 | 0.24 | 0.27 |
| SF | Day 3 A | 58.37 | 1.12 | 2.06 | 247.48 | 0.60 | 0.32 | 94.81 | 57.03 | 4.75 | 1.45 | 0.77 | 0.24 | 0.54 |
| SF | Day 3 B | 55.11 | 1.10 | 2.02 | 244.99 | 0.22 | 0.18 | 85.77 | 55.65 | 2.82 | 1.60 | 0.36 | 0.11 | 0.25 |
| SP | Day 3 A | 59.07 | 1.09 | 2.02 | 243.50 | 0.87 | 0.38 | 91.57 | 52.63 | 5.48 | 2.42 | 0.94 | 0.45 | 2.99 |
| SP | Day 3 B | 54.47 | 1.04 | 2.04 | 243.70 | 0.46 | 0.18 | 98.10 | 56.56 | 3.26 | 2.15 | 0.42 | 0.30 | 1.61 |
| LC | Day 3 A | 59.03 | 1.16 | 2.02 | 241.61 | 0.64 | 0.31 | 84.84 | 55.23 | 4.77 | 1.70 | 0.81 | 0.25 | 0.45 |
| LC | Day 3 B | 54.73 | 1.14 | 2.13 | 251.51 | 0.26 | 0.21 | 88.51 | 56.74 | 3.17 | 1.78 | 0.38 | 0.14 | 0.30 |
| LF | Day 3 A | 58.68 | 1.12 | 2.04 | 245.49 | 0.81 | 2.35 | 94.84 | 56.61 | 5.64 | 2.34 | 0.95 | 0.56 | 0.57 |
| LF | Day 3 B | 55.14 | 1.08 | 2.02 | 243.42 | 0.33 | 0.32 | 89.70 | 54.17 | 3.15 | 2.10 | 0.45 | 0.26 | 0.32 |
| LP | Day 3 A | 59.77 | 1.14 | 2.04 | 248.95 | 0.92 | 0.32 | 96.56 | 56.01 | 5.15 | 2.21 | 0.82 | 0.41 | 2.68 |
| LP | Day 3 B | 57.51 | 1.19 | 2.00 | 241.72 | 0.30 | 0.21 | 92.91 | 52.80 | 3.17 | 1.70 | 0.43 | 0.19 | 1.64 |
| SC | Day 5 | 52.70 | 1.07 | 2.06 | 248.20 | 0.17 | 0.20 | 84.34 | 54.71 | 2.83 | 1.44 | 0.36 | 0.12 | 0.22 |
| SL | Day 5 | 54.83 | 1.06 | 1.98 | 237.62 | 0.13 | 0.21 | 95.28 | 54.23 | 2.71 | 1.54 | 0.35 | 0.28 | 0.33 |
| SP | Day 5 | 53.48 | 1.11 | 2.00 | 240.19 | 0.28 | 0.22 | 89.83 | 54.59 | 2.92 | 1.79 | 0.45 | 0.19 | 1.55 |
| LC | Day 17 | 29.29 | 0.69 | 1.80 | 224.12 | 35.81 | 83.84 | 75.62 | 51.64 | 0.85 | 1.09 | 57.06 | 61.78 | 0.10 |
| LF | Day 17 | 30.33 | 0.67 | 1.70 | 212.77 | 35.98 | 86.21 | 67.59 | 44.59 | 0.97 | 0.95 | 46.87 | 59.60 | 0.12 |
| LP | Day 17 | 31.73 | 0.66 | 1.73 | 212.02 | 22.42 | 80.29 | 68.41 | 46.18 | 1.30 | 1.47 | 89.19 | 46.05 | 0.50 |
| LC | Day 29 | 31.29 | 0.74 | 1.82 | 226.05 | 33.17 | 77.05 | 67.98 | 49.25 | 1.23 | 1.07 | 48.41 | 90.30 | 0.12 |
| LF | Day 29 | 29.79 | 0.72 | 1.83 | 226.02 | 37.87 | 70.67 | 67.73 | 44.85 | 1.27 | 1.10 | 57.26 | 104.99 | 0.34 |
| LP | Day 29 | 32.84 | 0.74 | 1.78 | 219.20 | 23.34 | 92.83 | 63.59 | 44.31 | 1.14 | 1.07 | 81.87 | 93.52 | 0.12 |
| seawater mean (n=4) |  | 45.90 | 0.95 | 1.90 | 229.04 | 4.78 | 0.58 | 79.25 | 51.47 | 1.24 | 1.81 | 2.79 | 13.58 | 0.08 |
| seawater sd |  | 10.43 | 0.18 | 0.18 | 20.25 | 9.51 | 1.11 | 12.47 | 6.76 | 0.21 | 0.20 | 5.56 | 26.95 | 0.02 |

|  |  | ICP-MS |  |  |  |  |  |  |  |  | TXRF |  |  |
| --- | --- | --- | --- | --- | --- | --- | --- | --- | --- | --- | --- | --- | --- |
| Treatment | Timepoint | Ni 60  (ug/L) | Cu 63 (ug/L) | Zn 66 (ug/L) | As 75  (ug/L) | Se 82 (ug/L) | Rb 85 (ug/L) | Sr 88 (mg/L) | Ba 138  (mg/L) | Pb 208 (ug/L) | Br (mg/L) | Cl (mg/L) | S  (mg/L) |
| SC | start | 5.12 | 2.57 | 7.93 | 7.01 | 29.61 | 38.65 | 2.26 | 0.12 | 2.24 | 1.09 | 77.53 | 111.38 |
| SF | start | 4.01 | 1.93 | 5.80 | 8.96 | 27.04 | 38.08 | 2.25 | 0.11 | 1.90 | 0.95 | 114.32 | 67.88 |
| SP | start | 3.94 | 2.53 | 5.56 | 9.85 | 23.70 | 37.89 | 2.26 | 0.11 | 2.32 | 0.95 | 135.74 | 75.53 |
| LC | start | 3.88 | 2.24 | 5.33 | 8.39 | 12.02 | 37.38 | 2.23 | 0.11 | 2.01 | 0.96 | 97.09 | 77.94 |
| LF | start | 3.31 | 2.33 | 8.23 | 10.85 | 19.27 | 38.53 | 2.29 | 0.12 | 2.30 | 0.92 | 93.74 | 81.45 |
| LP | start | 3.85 | 2.57 | 6.30 | 10.08 | 12.77 | 38.90 | 2.24 | 0.12 | 2.10 | 1.03 | 121.55 | 92.65 |
| SC | Day 3 A | 3.36 | 2.08 | 6.45 | 7.31 | 17.65 | 38.58 | 2.29 | 0.11 | 1.16 | 0.78 | 84.00 | 49.26 |
| SC | Day 3 B | 3.14 | 2.33 | 7.07 | 2.88 | 12.02 | 33.61 | 2.14 | 0.07 | 0.60 | 0.79 | 77.90 | 68.30 |
| SF | Day 3 A | 3.17 | 1.70 | 17.50 | 7.33 | 11.74 | 34.49 | 2.27 | 0.10 | 0.81 | 0.82 | 67.21 | 107.70 |
| SF | Day 3 B | 3.13 | 2.56 | 6.84 | 5.14 | 11.17 | 34.43 | 2.20 | 0.07 | 0.50 | 0.82 | 111.89 | 59.56 |
| SP | Day 3 A | 3.82 | 5.04 | 692.41 | 8.99 | 20.48 | 36.14 | 2.27 | 0.11 | 1.58 | 0.84 | 165.16 | 80.70 |
| SP | Day 3 B | 3.27 | 7.20 | 378.94 | 5.80 | 19.64 | 33.61 | 2.18 | 0.07 | 0.86 | 0.94 | 73.70 | 59.86 |
| LC | Day 3 A | 3.32 | 2.06 | 2.56 | 8.62 | 25.75 | 36.56 | 2.27 | 0.10 | 0.80 | 1.00 | 43.32 | 93.44 |
| LC | Day 3 B | 3.23 | 2.79 | 5.51 | 4.59 | 12.84 | 34.33 | 2.19 | 0.07 | 0.58 | 0.70 | 42.95 | 74.32 |
| LF | Day 3 A | 3.44 | 2.16 | 7.31 | 11.33 | 23.45 | 37.54 | 2.30 | 0.11 | 1.05 | 0.84 | 40.24 | 69.97 |
| LF | Day 3 B | 3.40 | 2.52 | 8.07 | 5.54 | 25.70 | 34.95 | 2.24 | 0.07 | 0.69 | 0.77 | 28.16 | 62.85 |
| LP | Day 3 A | 3.50 | 6.79 | 705.95 | 7.43 | 22.66 | 36.45 | 2.26 | 0.11 | 1.24 | 0.97 | 121.10 | 62.91 |
| LP | Day 3 B | 4.00 | 6.17 | 430.77 | 4.31 | 19.01 | 35.15 | 2.30 | 0.07 | 0.72 | 0.76 | 77.50 | 73.19 |
| SC | Day 5 | 3.08 | 2.01 | 7.87 | 4.17 | 13.30 | 33.73 | 2.23 | 0.07 | 0.30 | 0.92 | 162.54 | 91.43 |
| SL | Day 5 | 2.95 | 2.01 | 7.50 | 3.45 | 21.20 | 33.94 | 2.21 | 0.07 | 0.27 | 0.87 | 58.81 | 63.75 |
| SP | Day 5 | 3.43 | 3.83 | 462.13 | 5.19 | 17.82 | 34.14 | 2.23 | 0.07 | 0.65 | 0.89 | 161.24 | 86.60 |
| LC | Day 17 | 1.88 | 1.66 | 13.76 | 1.88 | 7.42 | 19.70 | 1.29 | 28.99 | 0.37 | 0.74 | 79.65 | 48.92 |
| LF | Day 17 | 1.89 | 1.47 | 17.92 | 1.75 | 12.85 | 19.38 | 1.28 | 29.83 | 0.38 | 0.61 | 75.24 | 54.33 |
| LP | Day 17 | 2.43 | 1.94 | 72.68 | 2.17 | 20.63 | 20.29 | 1.30 | 28.99 | 0.55 | 0.65 | 92.39 | 57.22 |
| LC | Day 29 | 2.52 | 1.69 | 18.16 | 1.31 | 9.13 | 19.32 | 1.32 | 27.56 | 0.13 | 0.73 | 17.30 | 62.86 |
| LF | Day 29 | 2.81 | 1.61 | 100.06 | 1.54 | 17.86 | 19.86 | 1.32 | 27.56 | 0.15 | 0.85 | 41.00 | 62.45 |
| LP | Day 29 | 2.34 | 1.72 | 26.84 | 1.48 | 13.76 | 20.24 | 1.34 | 28.61 | 0.13 | 0.87 | 29.02 | 47.00 |
| seawater mean (n=4) |  | 3.03 | 2.52 | 16.28 | 1.71 | 20.85 | 29.14 | 1.92 | 6.27 | 0.22 | 0.70 | 60.33 | 60.21 |
| seawater sd |  | 0.18 | 0.91 | 9.59 | 0.41 | 6.71 | 6.18 | 0.41 | 12.45 | 0.16 | 0.08 | 42.96 | 21.13 |

**Table S3.** PAH concentrations in the treatment waters and seawater control at different time points (SC = short-term control, SF = short-term filtrate, SP = short-term particle, LC = long-term control, LF = long-term filtrate, LP = long-term particle). At the day 3, sample A has been taken prior to water renewal and sample B after that. PAHs presented in the table: naphthalene (NP), 2-methylnaphthalene (2-MNP), 1-methylnaphthalene (1-MNP), biphenyl (BP), 2,6-dimethylnpahtalene (2,6-DMN), acenaphthylene (ACY), acenaphthene (ACE), 2,3,5-trimethylnapthalene (2,3,5-TMN), fluorene (FLU), phenanthrene (PHE), anthracene (ANT), 1-methylphenanthrene (1-MPH), fluoranthene (FLA), pyrene (PYR), benzo[a]anthracene (BaA), chrysene (CHR), benzo[b]fluoranthene (BbF), benzo[k]fluoranthene (BkF), benzo[e]pyrene (BeP), benzo[a]pyrene (BaP), perylene (PER), indeno[1,2,3-cd]pyrene (IcdP), dibenzo[a,h]anthracene (DahA) and benzo[g,h,i]perylene (BghiP).

| **Treatment** | SC | SF | SP | LC | LF | LP | SC | SF | SP | LC | LF | LP |
| --- | --- | --- | --- | --- | --- | --- | --- | --- | --- | --- | --- | --- |
| **Timepoint** | Day 3 A | Day 3 A | Day 3 A | Day 3 A | Day 3 A | Day 3 A | Day 3 B | Day 3 B | Day 3 B | Day 3 B | Day 3 B | Day 3 B |
| **NP (µg/L)** | <0.02 | <0.02 | <0.02 | <0.02 | <0.02 | <0.02 | <0.02 | <0.02 | <0.02 | <0.02 | <0.02 | <0.02 |
| **2-MNP (µg/L)** | <0.02 | <0.02 | <0.02 | <0.02 | <0.02 | <0.02 | <0.02 | <0.02 | <0.02 | <0.02 | <0.02 | <0.02 |
| **1-MNP (µg/L)** | <0.02 | <0.02 | <0.02 | <0.02 | <0.02 | <0.02 | <0.02 | <0.02 | <0.02 | <0.02 | <0.02 | <0.02 |
| **BP (µg/L)** | <0.02 | <0.02 | <0.02 | <0.02 | <0.02 | <0.02 | <0.02 | <0.02 | <0.02 | <0.02 | <0.02 | <0.02 |
| **2,6-DMN (µg/L)** | <0.02 | <0.02 | 0.022 | <0.02 | <0.02 | <0.02 | <0.02 | <0.02 | <0.02 | <0.02 | <0.02 | <0.02 |
| **ACY (µg/L)** | <0.01 | <0.01 | 0.100 | <0.01 | <0.01 | 0.081 | <0.01 | <0.01 | 0.065 | <0.01 | <0.01 | 0.064 |
| **ACE (µg/L)** | <0.01 | 0.050 | <0.01 | <0.01 | <0.01 | <0.01 | <0.01 | <0.01 | <0.01 | <0.01 | <0.01 | <0.01 |
| **2,3,5-TMN (µg/L)** | <0.01 | <0.01 | 0.019 | <0.01 | <0.01 | 0.022 | <0.01 | <0.01 | 0.017 | <0.01 | <0.01 | 0.016 |
| **FLU (µg/L)** | <0.01 | <0.01 | 0.036 | <0.01 | <0.01 | 0.036 | <0.01 | <0.01 | 0.026 | <0.01 | <0.01 | 0.026 |
| **PHE (µg/L)** | <0.02 | <0.02 | 0.042 | <0.02 | <0.02 | 0.059 | <0.02 | <0.02 | 0.035 | <0.02 | <0.02 | 0.038 |
| **ANT (µg/L)** | <0.02 | <0.02 | <0.02 | <0.02 | <0.02 | <0.02 | <0.02 | <0.02 | <0.02 | <0.02 | <0.02 | <0.02 |
| **1-MPH (µg/L)** | <0.02 | <0.02 | <0.02 | <0.02 | <0.02 | <0.02 | <0.02 | <0.02 | <0.02 | <0.02 | <0.02 | <0.02 |
| **FLA (µg/L)** | <0.02 | <0.02 | 0.087 | <0.02 | <0.02 | 0.120 | <0.02 | <0.02 | 0.097 | <0.02 | <0.02 | 0.098 |
| **PYR (µg/L)** | <0.01 | <0.01 | 0.220 | <0.01 | <0.01 | 0.300 | <0.01 | <0.01 | 0.250 | <0.01 | <0.01 | 0.270 |
| **BaA (µg/L)** | <0.01 | <0.01 | <0.01 | <0.01 | <0.01 | <0.01 | <0.01 | <0.01 | <0.01 | <0.01 | <0.01 | <0.01 |
| **CHR (µg/L)** | <0.01 | <0.01 | <0.01 | <0.01 | <0.01 | 0.013 | <0.01 | <0.01 | 0.012 | <0.01 | <0.01 | <0.01 |
| **BbF (µg/L)** | <0.01 | <0.01 | <0.01 | <0.01 | <0.01 | <0.01 | <0.01 | <0.01 | 0.010 | <0.01 | <0.01 | <0.01 |
| **BkF (µg/L)** | <0.01 | <0.01 | <0.01 | <0.01 | <0.01 | <0.01 | <0.01 | <0.01 | 0.010 | <0.01 | <0.01 | <0.01 |
| **BeP (µg/L)** | <0.01 | <0.01 | <0.01 | <0.01 | <0.01 | 0.014 | <0.01 | <0.01 | 0.012 | <0.01 | <0.01 | 0.012 |
| **BaP (µg/L)** | 0.004 | <0.002 | 0.008 | <0.002 | 0.004 | 0.011 | <0.002 | <0.002 | 0.009 | <0.002 | <0.002 | 0.009 |
| **PER (µg/L)** | <0.01 | <0.01 | <0.01 | <0.01 | <0.01 | <0.01 | <0.01 | <0.01 | <0.01 | <0.01 | <0.01 | <0.01 |
| **IcdP (µg/L)** | 0.011 | <0.01 | 0.021 | <0.01 | 0.012 | 0.021 | <0.01 | <0.01 | 0.018 | <0.01 | <0.01 | 0.015 |
| **DahA (µg/L)** | <0.01 | <0.01 | <0.01 | <0.01 | <0.01 | <0.01 | <0.01 | <0.01 | <0.01 | <0.01 | <0.01 | <0.01 |
| **BghiP (µg/L)** | 0.007 | 0.005 | 0.031 | <0.002 | 0.007 | 0.042 | 0.002 | <0.002 | 0.045 | <0.002 | 0.004 | 0.046 |
| **PAHsum (µg/L)** | <0.1 | <0.1 | 0.590 | <0.1 | <0.1 | 0.720 | <0.1 | <0.1 | 0.590 | <0.1 | <0.1 | 0.590 |

| **Treatment** | SC | SF | SP | LC | LF | LP |
| --- | --- | --- | --- | --- | --- | --- |
| **Timepoint** | Day 5 | Day 5 | Day 5 | Day 29 | Day 29 | Day 29 |
| **NP (µg/L)** | <0.02 | <0.02 | <0.02 | <0.02 | <0.02 | <0.02 |
| **2-MNP (µg/L)** | <0.02 | <0.02 | <0.02 | <0.02 | <0.02 | <0.02 |
| **1-MNP (µg/L)** | <0.02 | <0.02 | <0.02 | <0.02 | <0.02 | <0.02 |
| **BP (µg/L)** | <0.02 | <0.02 | <0.02 | <0.02 | <0.02 | <0.02 |
| **2,6-DMN (µg/L)** | <0.02 | <0.02 | <0.02 | <0.02 | <0.02 | <0.02 |
| **ACY (µg/L)** | <0.01 | <0.01 | 0.077 | <0.01 | <0.01 | 0.023 |
| **ACE (µg/L)** | <0.01 | <0.01 | <0.01 | <0.01 | <0.01 | <0.01 |
| **2,3,5-TMN (µg/L)** | <0.01 | <0.01 | <0.01 | <0.01 | <0.01 | <0.01 |
| **FLU (µg/L)** | <0.01 | <0.01 | 0.029 | 0.012 | 0.012 | 0.021 |
| **PHE (µg/L)** | <0.02 | <0.02 | 0.033 | <0.02 | <0.02 | <0.02 |
| **ANT (µg/L)** | <0.02 | <0.02 | <0.02 | <0.02 | <0.02 | <0.02 |
| **1-MPH (µg/L)** | <0.02 | <0.02 | <0.02 | <0.02 | <0.02 | <0.02 |
| **FLA (µg/L)** | <0.02 | <0.02 | 0.054 | <0.02 | <0.02 | <0.02 |
| **PYR (µg/L)** | <0.01 | <0.01 | 0.130 | 0.014 | <0.01 | 0.030 |
| **BaA (µg/L)** | <0.01 | <0.01 | <0.01 | <0.01 | <0.01 | <0.01 |
| **CHR (µg/L)** | <0.01 | <0.01 | <0.01 | <0.01 | <0.01 | <0.01 |
| **BbF (µg/L)** | <0.01 | <0.01 | <0.01 | <0.01 | <0.01 | <0.01 |
| **BkF (µg/L)** | <0.01 | <0.01 | <0.01 | <0.01 | <0.01 | <0.01 |
| **BeP (µg/L)** | <0.01 | <0.01 | <0.01 | <0.01 | <0.01 | <0.01 |
| **BaP (µg/L)** | <0.002 | <0.002 | 0.003 | 0.003 | <0.002 | <0.002 |
| **PER (µg/L)** | <0.01 | <0.01 | <0.01 | <0.01 | <0.01 | <0.01 |
| **IcdP (µg/L)** | <0.01 | <0.01 | 0.011 | <0.01 | <0.01 | <0.01 |
| **DahA (µg/L)** | <0.01 | <0.01 | <0.01 | <0.01 | <0.01 | <0.01 |
| **BghiP (µg/L)** | <0.002 | <0.002 | 0.018 | <0.002 | <0.002 | <0.002 |
| **PAHsum (µg/L)** | <0.1 | <0.1 | 0.360 | <0.1 | <0.1 | <0.1 |

**Table S4.** Metal concentrations in the clams at the end of the experiment (SC = short-term control, SF = short-term filtrate, SP = short-term particle, LC = long-term control, LF = long-term filtrate, LP = long-term particle).

| **Treatment** | **Replicate** | **As (mg/kg)** | **Cd (mg/kg)** | **Co (mg/kg)** | **Cr (mg/kg)** | **Cu (mg/kg)** | **Fe (mg/kg)** | **Ni (mg/kg)** | **Pb (mg/kg)** | **Se (mg/kg)** | **U* (mg/kg)** | **V (mg/kg)** | **Zn (mg/kg)** |
| --- | --- | --- | --- | --- | --- | --- | --- | --- | --- | --- | --- | --- | --- |
| SC | A | 11 | 0.74 | 4.6 | 1.3 | 498 | 1245 | 2.0 | 2.9 | 3.1 | 0.86 | 1.5 | 942 |
| SC | B | 12 | 0.54 | 4.4 | 1.2 | 672 | 1271 | 2.0 | 3.0 | 3.5 | 0.94 | 1.5 | 981 |
| SF | A | 13 | 0.60 | 5.1 | 1.4 | 574 | 1525 | 2.0 | 3.1 | 3.7 | 0.94 | 1.8 | 1081 |
| SF | B | 12 | 0.74 | 4.5 | 1.4 | 639 | 1598 | 2.2 | 3.0 | 3.7 | 1.0 | 1.7 | 1013 |
| SP | A | 10 | 0.68 | 9.6 | 1.4 | 594 | 1480 | 1.9 | 4.1 | 3.2 | 0.77 | 1.6 | 1383 |
| SP | B | 11 | 0.83 | 11 | 1.2 | 639 | 1102 | 1.7 | 3.0 | 3.3 | 0.82 | 1.4 | 1715 |
| LC | A | 13 | 0.80 | 6.5 | 1.9 | 571 | 1686 | 2.7 | 3.6 | 3.3 | 1.2 | 2.1 | 1247 |
| LC | B | 11 | 0.70 | 5.8 | 1.8 | 532 | 1432 | 2.8 | 3.2 | 3.7 | 1.2 | 1.9 | 1129 |
| LF | A | 10 | 0.69 | 6.0 | 1.6 | 591 | 1528 | 2.5 | 3.0 | 3.0 | 0.90 | 1.9 | 1375 |
| LF | B | 10 | 0.58 | 3.6 | 1.3 | 477 | 1264 | 2.4 | 2.7 | 2.8 | 0.84 | 1.5 | 933 |
| LP | A | 11 | 0.85 | 11 | 1.2 | 547 | 970 | 2.5 | 3.2 | 3.2 | 0.86 | 1.3 | 1540 |
| LP | B | 10 | 0.59 | 13 | 1.5 | 563 | 1518 | 2.2 | 3.4 | 3.0 | 0.84 | 1.7 | 1570 |
| Method accredited |  | yes | yes | yes | yes | yes | no | yes | yes | yes | no | yes | yes |
| *results not verified with reference material | | | | | | | | | | | | | |

**Table S5.** Polycyclic aromatic hydrocarbon (PAH) concentrations in the clams at the end of the experiment (SC = short-term control, SF = short-term filtrate, SP = short-term particle, LC = long-term control, LF = long-term filtrate, LP = long-term particle). PAHs presented in the table_ naphthalene (NP), 2-methylnaphthalene (2-MNP), 1-methylnaphthalene (1-MNP), biphenyl (BP), 2,6-dimethylnpahtalene (2,6-DMN), acenaphthylene (ACY), acenaphthene (ACE), 2,3,5-trimethylnapthalene (2,3,5-TMN), fluorene (FLU), phenanthrene (PHE), anthracene (ANT), 1-methylphenanthrene (1-MPH), fluoranthene (FLA), pyrene (PYR), benzo[a]anthracene (BaA), chrysene (CHR), benzo[b]fluoranthene (BbF), benzo[k]fluoranthene (BkF), benzo[e]pyrene (BeP), benzo[a]pyrene (BaP), perylene (PER), indeno[1,2,3-cd]pyrene (IcdP), dibenzo[a,h]anthracene (DahA) and benzo[g,h,i]perylene (BghiP).

| **Treatment** | SC | SC | SC | SF | SF | SF | SP | SP | SP | LC | LC | LF | LF | LP | LP |
| --- | --- | --- | --- | --- | --- | --- | --- | --- | --- | --- | --- | --- | --- | --- | --- |
| **Replicate** | A | B | C | A | B | C | A | B | C | A | B | A | B | A | B |
| **NP (µg/kg)** | 69 | 71 | 57 | 39 | 54 | 63 | 54 | 51 | 77 | 14 | 19 | 15 | 21 | 26 | 15 |
| **2-MNP (µg/kg)** | 56 | 70 | 54 | 32 | 46 | 52 | 59 | 53 | 82 | 13 | 20 | 14 | 26 | 25 | 14 |
| **1-MNP (µg/kg)** | 25 | 29 | 23 | 14 | 20 | 24 | 29 | 29 | 46 | 6.1 | 8.6 | 6.1 | 11 | 11 | 6.6 |
| **ACY (µg/kg)** | 4.0 | 5.6 | 4.4 | 4.4 | 5.2 | 5.4 | 37 | 40 | 39 | 2.2 | <1 | 1.3 | 1.5 | 7.5 | 6.6 |
| **ACE (µg/kg)** | <1 | <1 | <1 | <1 | <1 | 1.5 | 11 | 11 | 11 | <1 | <1 | <1 | <1 | 3.0 | 3.7 |
| **FLU (µg/kg)** | <1 | <1 | <1 | <1 | <1 | 2.3 | 11 | 14 | 12 | <1 | <1 | <1 | <1 | 7.3 | 7.1 |
| **PHE (µg/kg)** | 3.9 | 5.2 | 3.3 | 3.8 | 5.4 | 5.0 | 75 | 84 | 72 | 1.9 | 2.1 | 2.3 | 2.3 | 60 | 63 |
| **ANT (µg/kg)** | 1.7 | <1 | 1.1 | <1 | <1 | <1 | 9.7 | 9.3 | 8.4 | <1 | <1 | 1.0 | <1 | 12 | 12 |
| **FLA (µg/kg)** | 12 | 9.4 | 4.6 | 8.5 | 9.3 | 12 | 130 | 120 | 100 | 6.2 | 6.5 | 5.7 | 5.9 | 160 | 160 |
| **PYR (µg/kg)** | 11 | 11 | 5.2 | 8.9 | 13 | 12 | 490 | 440 | 410 | 6.8 | 7.1 | 5.4 | 5.9 | 650 | 630 |
| **BaA (µg/kg)** | 8.2 | 8.2 | 7.5 | 7.0 | 9.6 | 8.3 | 9.5 | 9.8 | 9.7 | 4.8 | 4.7 | 4.5 | 4.2 | 6.4 | 7.2 |
| **TRI (µg/kg)** | 12 | 14 | 15 | 11 | 15 | 11 | 20 | 20 | 19 | 8.6 | 8.7 | 8.1 | 7.1 | 20 | 18 |
| **CHR (µg/kg)** | 6.4 | 6.9 | 6.4 | 4.8 | 7.6 | 6.8 | 11 | 11 | 12 | 2.3 | 2.2 | 2.1 | 2.4 | 8.9 | 9.7 |
| **BbF (µg/kg)** | 13 | 14 | 9.2 | 9.3 | 12 | 14 | 11 | 11 | 12 | 9.6 | 9.8 | 9.3 | 8.3 | 7.1 | 8.0 |
| **BkF (µg/kg)** | 4.5 | 4.4 | 3.1 | 3.2 | 2.6 | 4.3 | 4.4 | <2 | 3.0 | 3.1 | 3.1 | 3.1 | 2.7 | 2.0 | <2 |
| **BeP (µg/kg)** | 12 | 15 | 5.8 | 5.9 | 8.2 | 10 | 16 | 16 | 18 | 9.0 | 10 | 10 | 8.2 | 14 | 16 |
| **BaP (µg/kg)** | 4.4 | 4.5 | <2 | <2 | 3.4 | 5.0 | 5.8 | 7.6 | 7.9 | 3.1 | 3.4 | 4.2 | 2.6 | 5.4 | 6.5 |
| **PER (µg/kg)** | 4.1 | <2 | <2 | <2 | <2 | <2 | 5.4 | 4.2 | 5.0 | <2 | <2 | 2.1 | <2 | <2 | <2 |
| **IcdP (µg/kg)** | <2 | <2 | <2 | 8.3 | 7.5 | 7.9 | 10 | 9.1 | 12 | 3.9 | 2.7 | 3.8 | 2.5 | 2.5 | <2 |
| **DahA (µg/kg)** | <2 | <2 | <2 | <2 | <2 | <2 | <2 | <2 | <2 | <2 | <2 | <2 | <2 | <2 | <2 |
| **BghiP (µg/kg)** | 21 | 27 | 8.3 | 13 | 14 | 19 | 62 | 55 | 68 | 6.7 | 7.5 | 7.4 | 6.2 | 20 | 23 |
| **Fat (%)** | 1.8 | 1.7 | 2.0 | 2.1 | 1.8 | 1.7 | 2.0 | 2.0 | 1.9 | 1.6 | 1.5 | 1.6 | 1.4 | 1.5 | 1.6 |

**Table S6.** Geno- and cytotoxity biomarkers analysed in clam gills in the different treatments at the end of the experiment (SC = short-term control, SF = short-term filtrate, SP = short-term particle, LC = long-term control, LF = long-term filtrate, LP = long-term particle). Bi-nucleated (BN) and 8-shaped nuclei cells describe cytotoxic effects (Cytox) and the rest represent genotoxic (Gentox) effects (BL = blebbed nuclei, BNb = bi-nucleated cells with nucleoplasmic bridges, MN = micronuclei, NB = nuclear buds, NBf = nuclear buds on filament).

| Treatment | Sample ID | MN | BNb | NBf | NB | BL | 8-shaped | BN | Gentox | Cytox | total cytogenetic damage |
| --- | --- | --- | --- | --- | --- | --- | --- | --- | --- | --- | --- |
| SC | G1 | 1 | 0 | 0 | 1 | 2.5 | 0.5 | 0 | 4.5 | 0.5 | 5 |
| SC | G2 | 1.5 | 0 | 0 | 2 | 1.5 | 0.5 | 0.5 | 5 | 1 | 6 |
| SC | G3 | 2 | 0 | 0 | 1.5 | 0.5 | 0 | 0 | 4 | 0 | 4 |
| SC | G4 | 1.5 | 0 | 0 | 1 | 1 | 1 | 0 | 3.5 | 1 | 4.5 |
| SC | G5 | 1 | 0 | 0 | 1 | 0.5 | 0 | 0 | 2.5 | 0 | 2.5 |
| SC | G6 | 1 | 0 | 0 | 2 | 0.5 | 0 | 0 | 3.5 | 0 | 3.5 |
| SC | G7 | 0.5 | 0 | 0 | 1.5 | 1 | 0 | 0 | 3 | 0 | 3 |
| SC | G8 | 0 | 0 | 0 | 0.5 | 0.5 | 0 | 0 | 1 | 0 | 1 |
| SC | G9 | 0 | 0 | 0 | 2 | 1 | 1 | 0 | 3 | 1 | 4 |
| SC | G10 | 1.5 | 0 | 0 | 1.5 | 0.5 | 0 | 0 | 3.5 | 0 | 3.5 |
| SC | G11 | 1 | 0 | 0 | 1 | 0.5 | 0 | 0 | 2.5 | 0 | 2.5 |
| SC | G12 | 0 | 0 | 0 | 1 | 1 | 0.5 | 0 | 2 | 0.5 | 2.5 |
| SC | G13 | 0 | 0 | 0 | 1.5 | 1.5 | 0 | 0 | 3 | 0 | 3 |
| SC | G14 | 2 | 0 | 0 | 1.5 | 1.5 | 1 | 0 | 5 | 1 | 6 |
| SC | G15 | 1 | 0 | 0 | 0.5 | 2 | 0 | 0 | 3.5 | 0 | 3.5 |
| SC | G16 | 0 | 0 | 0 | 0.5 | 0 | 1 | 0 | 0.5 | 1 | 1.5 |
| SC | G17 | 0.5 | 0 | 0 | 1 | 1.5 | 0.5 | 0 | 3 | 0.5 | 3.5 |
| SC | G18 | 1 | 0 | 0.5 | 2 | 1 | 1 | 0 | 4.5 | 1 | 5.5 |
| SC | G19 | 0.5 | 0 | 0 | 1 | 1.5 | 0.5 | 0 | 3 | 0.5 | 3.5 |
| SC | G20 | 0.5 | 0 | 0 | 0.5 | 1.5 | 0 | 0 | 2.5 | 0 | 2.5 |
| SF | G1 | 0.5 | 0 | 0 | 1.5 | 1 | 0.5 | 0 | 3 | 0.5 | 3.5 |
| SF | G2 | 0.5 | 0 | 0 | 0.5 | 2.5 | 0 | 0 | 3.5 | 0 | 3.5 |
| SF | G3 | 0 | 0 | 0 | 0.5 | 1.5 | 0 | 0 | 2 | 0 | 2 |
| SF | G4 | 1 | 0 | 0.5 | 1.5 | 1.5 | 0 | 0 | 4.5 | 0 | 4.5 |
| SF | G5 | 0 | 0 | 0.5 | 0.5 | 1 | 0 | 0 | 2 | 0 | 2 |
| SF | G6 | 0.5 | 0 | 0 | 1 | 0.5 | 0 | 0 | 2 | 0 | 2 |
| SF | G7 | 0 | 0 | 0 | 1 | 0 | 0 | 0 | 1 | 0 | 1 |
| SF | G8 | 0.5 | 0 | 0.5 | 1 | 0.5 | 0 | 0 | 2.5 | 0 | 2.5 |
| SF | G9 | 1 | 0 | 0 | 1.5 | 1 | 0.5 | 0 | 3.5 | 0.5 | 4 |
| SF | G10 | 1 | 0 | 0 | 1.5 | 0.5 | 0 | 0 | 3 | 0 | 3 |
| SF | G11 | 0.5 | 0 | 0 | 2 | 1 | 0.5 | 0 | 3.5 | 0.5 | 4 |
| SF | G12 | 2 | 0 | 0 | 1 | 1.5 | 0 | 0.5 | 4.5 | 0.5 | 5 |
| SF | G13 | 1 | 0 | 0 | 0.5 | 1 | 0.5 | 0 | 2.5 | 0.5 | 3 |
| SF | G14 | 0.5 | 0 | 0 | 1 | 1 | 0 | 0 | 2.5 | 0 | 2.5 |
| SF | G15 | 0.5 | 0 | 0 | 1 | 0.5 | 0 | 0 | 2 | 0 | 2 |
| SF | G16 | 1 | 0 | 0 | 0 | 0.5 | 0 | 0 | 1.5 | 0 | 1.5 |
| SF | G17 | 0 | 0 | 0 | 1.5 | 1 | 0.5 | 0 | 2.5 | 0.5 | 3 |
| SF | G18 | 1 | 0 | 0 | 1.5 | 1 | 0 | 0 | 3.5 | 0 | 3.5 |
| SF | G19 | 1 | 0.5 | 0 | 1 | 1 | 0.5 | 0 | 3.5 | 0.5 | 4 |
| SF | G20 | 1 | 0 | 0 | 0.5 | 1 | 0 | 0 | 2.5 | 0 | 2.5 |
| SP | G1 | 0 | 0 | 0 | 1 | 1.5 | 0 | 0 | 2.5 | 0 | 2.5 |
| SP | G2 | 2 | 0 | 0 | 2 | 2 | 0 | 0 | 6 | 0 | 6 |
| SP | G3 | 1.5 | 0 | 0 | 0.5 | 3 | 0 | 0 | 5 | 0 | 5 |
| SP | G4 | 0 | 0 | 0 | 1 | 2.5 | 0.5 | 0 | 3.5 | 0.5 | 4 |
| SP | G5 | 0.5 | 0 | 0 | 1 | 1 | 0.5 | 0 | 2.5 | 0.5 | 3 |
| SP | G6 | 0 | 0 | 0 | 0.5 | 1.5 | 0 | 0 | 2 | 0 | 2 |
| SP | G7 | 0.5 | 0 | 0 | 1.5 | 1.5 | 0 | 0 | 3.5 | 0 | 3.5 |
| SP | G8 | 0 | 0 | 0 | 0.5 | 0.5 | 0.5 | 0 | 1 | 0.5 | 1.5 |
| SP | G9 | 1 | 0 | 0 | 1.5 | 0.5 | 0 | 0 | 3 | 0 | 3 |
| SP | G10 | 0.5 | 0 | 0 | 1 | 1.5 | 1 | 0 | 3 | 1 | 4 |
| SP | G11 | 0 | 0 | 0 | 1 | 1.5 | 0.5 | 0 | 2.5 | 0.5 | 3 |
| SP | G12 | 0.5 | 0 | 0 | 1 | 1 | 0 | 0 | 2.5 | 0 | 2.5 |
| SP | G13 | 0.5 | 0 | 0 | 1.5 | 1.5 | 0.5 | 0 | 3.5 | 0.5 | 4 |
| SP | G14 | 1 | 0 | 0 | 2.5 | 1.5 | 1 | 0 | 5 | 1 | 6 |
| SP | G15 | 1 | 0 | 0 | 0.5 | 0.5 | 0 | 0 | 2 | 0 | 2 |
| SP | G16 | 0 | 0 | 0 | 0 | 0 | 0 | 0 | 0 | 0 | 0 |
| SP | G17 | 0.5 | 0 | 0 | 1.5 | 0.5 | 0.5 | 0 | 2.5 | 0.5 | 3 |
| SP | G18 | 0 | 0 | 0 | 2 | 1.5 | 0 | 0 | 3.5 | 0 | 3.5 |
| SP | G19 | 1 | 0 | 0 | 2 | 0.5 | 0.5 | 0 | 3.5 | 0.5 | 4 |
| SP | G20 | 1 | 0 | 0 | 1 | 0.5 | 0.5 | 0 | 2.5 | 0.5 | 3 |
| LC | G1 | 1 | 0 | 0 | 1 | 1 | 0.5 | 0.5 | 3 | 1 | 4 |
| LC | G2 | 1 | 0 | 0 | 1.5 | 3 | 0 | 0 | 5.5 | 0 | 5.5 |
| LC | G3 | 0.5 | 0 | 0 | 1.5 | 1.5 | 0 | 0 | 3.5 | 0 | 3.5 |
| LC | G4 | 1 | 0 | 0 | 2.5 | 0.5 | 0 | 0 | 4 | 0 | 4 |
| LC | G5 | 0.5 | 0 | 0.5 | 0.5 | 1.5 | 0 | 0 | 3 | 0 | 3 |
| LC | G6 | 1 | 0 | 0 | 2 | 2.5 | 0 | 0 | 5.5 | 0 | 5.5 |
| LC | G7 | 0 | 0 | 0 | 2 | 1 | 0 | 0 | 3 | 0 | 3 |
| LC | G8 | 0.5 | 0 | 0 | 1.5 | 0 | 0 | 0 | 2 | 0 | 2 |
| LC | G9 | 1 | 0 | 0.5 | 2 | 2 | 1 | 0 | 5.5 | 1 | 6.5 |
| LC | G10 | 0 | 0 | 0 | 1.5 | 1 | 0 | 0 | 2.5 | 0 | 2.5 |
| LC | G11 | 0 | 0 | 0 | 0.5 | 0.5 | 0 | 0 | 1 | 0 | 1 |
| LC | G12 | 0.5 | 0 | 0 | 1 | 1.5 | 0 | 0 | 3 | 0 | 3 |
| LC | G13 | 0.5 | 0 | 0 | 1 | 0 | 0.5 | 0 | 1.5 | 0.5 | 2 |
| LC | G14 | 1 | 0 | 0 | 1.5 | 0.5 | 0.5 | 0 | 3 | 0.5 | 3.5 |
| LC | G15 | 1 | 0 | 0.5 | 1.5 | 1 | 0.5 | 0 | 4 | 0.5 | 4.5 |
| LC | G16 | 0 | 0 | 0 | 0.5 | 0 | 0 | 0 | 0.5 | 0 | 0.5 |
| LC | G17 | 0.5 | 0 | 0 | 1 | 2 | 0 | 0 | 3.5 | 0 | 3.5 |
| LC | G18 | 0.5 | 0 | 0 | 1 | 1.5 | 0.5 | 0 | 3 | 0.5 | 3.5 |
| LC | G19 | 0 | 0 | 0 | 1.5 | 0.5 | 0.5 | 0 | 2 | 0.5 | 2.5 |
| LC | G20 | 0.5 | 0 | 0.5 | 0.5 | 1 | 0 | 0 | 2.5 | 0 | 2.5 |
| LF | G1 | 0 | 0 | 0 | 1 | 1.5 | 0 | 0 | 2.5 | 0 | 2.5 |
| LF | G2 | 1 | 0 | 0 | 2 | 0.5 | 0 | 0 | 3.5 | 0 | 3.5 |
| LF | G3 | 0.5 | 0 | 0 | 2.5 | 0.5 | 0 | 0 | 3.5 | 0 | 3.5 |
| LF | G4 | 2 | 0 | 0.5 | 1 | 1.5 | 0.5 | 0.5 | 5 | 1 | 6 |
| LF | G5 | 1 | 0 | 0.5 | 1.5 | 1.5 | 1.5 | 0 | 4.5 | 1.5 | 6 |
| LF | G6 | 0 | 0 | 0 | 1 | 2 | 0.5 | 0 | 3 | 0.5 | 3.5 |
| LF | G7 | 1.5 | 0 | 0.5 | 1 | 1.5 | 0.5 | 0.5 | 4.5 | 1 | 5.5 |
| LF | G8 | 1 | 0 | 0 | 1 | 1 | 0 | 0 | 3 | 0 | 3 |
| LF | G9 | 1.5 | 0 | 1 | 2.5 | 3 | 0.5 | 0 | 8 | 0.5 | 8.5 |
| LF | G10 | 1 | 0 | 0 | 2 | 1.5 | 0 | 0 | 4.5 | 0 | 4.5 |
| LF | G11 | 0.5 | 0 | 0 | 1 | 1 | 0.5 | 0 | 2.5 | 0.5 | 3 |
| LF | G12 | 1 | 0 | 0.5 | 2 | 1 | 0 | 0 | 4.5 | 0 | 4.5 |
| LF | G13 | 0.5 | 0 | 0.5 | 2.5 | 1.5 | 0.5 | 0 | 5 | 0.5 | 5.5 |
| LF | G14 | 1.5 | 0 | 1 | 3.5 | 2 | 1 | 0 | 8 | 1 | 9 |
| LF | G15 | 0.5 | 0 | 0 | 2 | 1.5 | 1 | 0 | 4 | 1 | 5 |
| LF | G16 | 1 | 0 | 0 | 1.5 | 2.5 | 0.5 | 0 | 5 | 0.5 | 5.5 |
| LF | G17 | 0.5 | 0 | 0 | 1.5 | 2.5 | 0.5 | 0 | 4.5 | 0.5 | 5 |
| LF | G18 | 0 | 0 | 0 | 1 | 0.5 | 0 | 0 | 1.5 | 0 | 1.5 |
| LF | G19 | 1 | 0 | 0 | 1.5 | 1 | 1 | 0 | 3.5 | 1 | 4.5 |
| LF | G20 | 1 | 0 | 0 | 1.5 | 0.5 | 0 | 0 | 3 | 0 | 3 |
| LP | G1 | 0.5 | 0 | 0 | 3 | 2 | 1 | 0 | 5.5 | 1 | 6.5 |
| LP | G2 | 1 | 0 | 0.5 | 0.5 | 1.5 | 0.5 | 0 | 3.5 | 0.5 | 4 |
| LP | G3 | 0.5 | 0 | 0 | 1 | 2 | 0 | 0 | 3.5 | 0 | 3.5 |
| LP | G4 | 1.5 | 0 | 0 | 2 | 1.5 | 0 | 0 | 5 | 0 | 5 |
| LP | G5 | 1 | 0 | 0 | 2 | 1 | 0.5 | 0 | 4 | 0.5 | 4.5 |
| LP | G6 | 0.5 | 0 | 0 | 2 | 2 | 0.5 | 0 | 4.5 | 0.5 | 5 |
| LP | G7 | 1.5 | 0 | 0.5 | 1.5 | 2.5 | 0 | 0 | 6 | 0 | 6 |
| LP | G8 | 1 | 0 | 0 | 1.5 | 1.5 | 1 | 0.5 | 4 | 1.5 | 5.5 |
| LP | G9 | 0.5 | 0 | 0 | 1 | 1.5 | 0.5 | 0 | 3 | 0.5 | 3.5 |
| LP | G10 | 1.5 | 0 | 0.5 | 2.5 | 2.5 | 1 | 0 | 7 | 1 | 8 |
| LP | G11 | 0 | 0 | 0 | 1 | 1 | 0 | 0 | 2 | 0 | 2 |
| LP | G12 | 1 | 0 | 0 | 1.5 | 1 | 0.5 | 0 | 3.5 | 0.5 | 4 |
| LP | G13 | 1.5 | 0 | 0.5 | 2 | 0 | 0 | 0 | 4 | 0 | 4 |
| LP | G14 | 1 | 0 | 0 | 1 | 1.5 | 0.5 | 0 | 3.5 | 0.5 | 4 |
| LP | G15 | 0.5 | 0 | 0 | 1 | 2 | 0.5 | 0 | 3.5 | 0.5 | 4 |
| LP | G16 | 1 | 0 | 0 | 2 | 2 | 0 | 0 | 5 | 0 | 5 |
| LP | G17 | 2 | 0 | 0.5 | 1 | 1 | 0.5 | 0 | 4.5 | 0.5 | 5 |
| LP | G18 | 1 | 0 | 0 | 1.5 | 0.5 | 0 | 0 | 3 | 0 | 3 |
| LP | G19 | 0.5 | 0 | 0 | 2.5 | 1 | 0.5 | 0 | 4 | 0.5 | 4.5 |
| LP | G20 | 0 | 0 | 0 | 1.5 | 0 | 0 | 0 | 1.5 | 0 | 1.5 |

**Table S7.** Deviations from normal cell structure, determined by visual inspection with a transmission electron microscope from the selected tissues (gills, DG = digestive gland, foot) of the clams (SC = short-term control, SF = short-term filtrate, SP = short-term particle, LC = long-term control, LF = long-term filtrate, LP = long-term particle). The anomalies are marked with x.

| Observed change compared to normal cell structure |  | SC | SF | SP | LC | LF | LP |
| --- | --- | --- | --- | --- | --- | --- | --- |
| Gills | swollen mitochondria |  | x |  |  | x | x |
|  | dark material inside lysosomes |  |  |  | x | x | x |
|  | enlarged lysosomes |  |  | x |  | x |  |
|  | increased number of lysosomes |  |  | x |  | x |  |
|  | polycyclic lysosomes | x |  |  |  |  |  |
|  | loosened cell junctions |  |  | x |  |  |  |
| DG | swollen mitochondria |  |  |  |  | x | x |
|  | uneven cilia in the epithelium |  |  | x | x | x | x |
|  | increased number of lysosomes |  |  |  | x |  | x |
| Foot | dark material inside lysosomes | x |  | x |  |  | x |
|  | increased number of lysosomes | x |  | x |  |  | x |
|  | decreased number of mitochondria | x |  | x |  |  |  |
|  | Total number of observed changes | 4 | 1 | 7 | 3 | 6 | 7 |


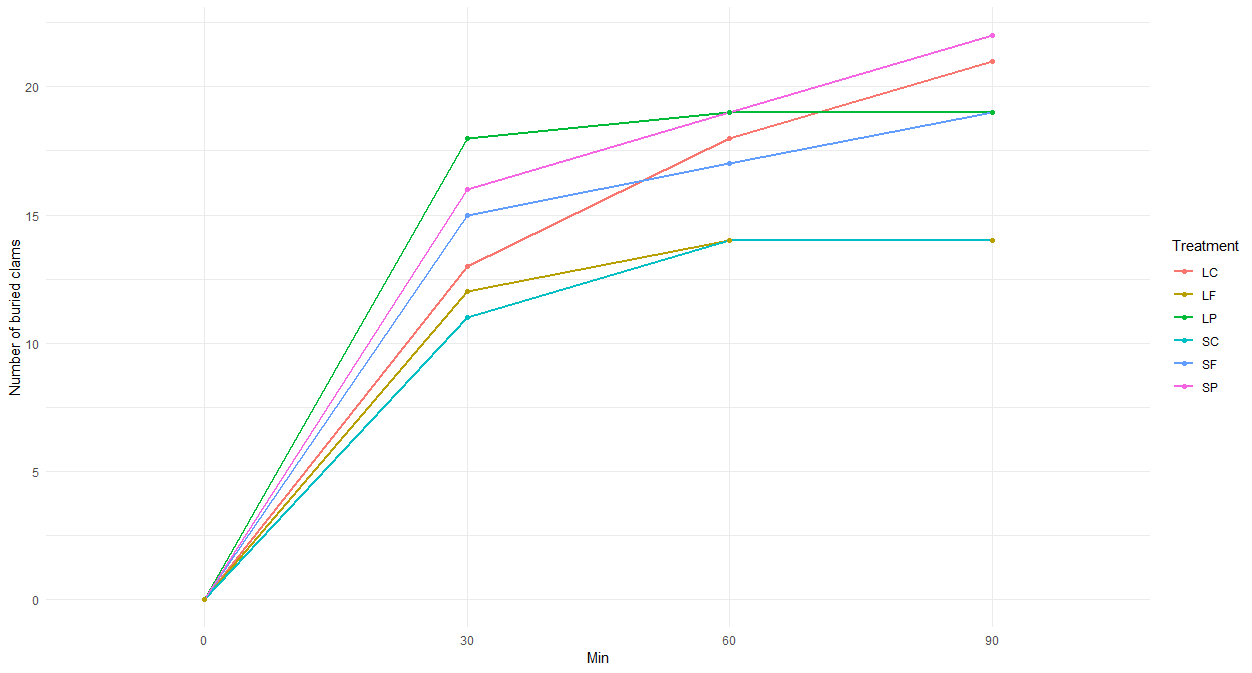


**Figure S2.** Number of *M. balthica* individuals burrowed in the clean sediment at 30-min intervals after ending the experiment (SC = short-term control, SF = short-term filtrate, SP = short-term particle, LC = long-term control, LF = long-term filtrate, LP = long-term particle). In the burrowing trial study, 25 clams from each treatment were randomly selected and placed on the surface of clean sediment in aquaria filled with fresh seawater (8.7–8.8 °C, dissolved oxygen 11.2–11.4 mg/L, and pH 7.96–8.02). During a 90-minute time period, the clams burrowed into sediment were counted every 30 minutes.
